# The Biogenetic Origin of the Biologically Active Naematolin of *Hypholoma* Species Involves an Unusual Sesquiterpene Synthase

**DOI:** 10.1007/s12033-019-00199-x

**Published:** 2019-08-07

**Authors:** Suhad A. A. Al-Salihi, Trong Tuan Dao, Katherine Williams, Andy M. Bailey, Gary D. Foster

**Affiliations:** 1 School of Biological Sciences, University of Bristol, 24 Tyndall Avenue, Bristol, BS8 1TQ UK; 2 School of Chemistry, University of Bristol, Cantock’s Close, Bristol, BS8 1TS UK

**Keywords:** *Hypholoma* species, Genome mining, Naematolin, Caryophyllene synthase

## Abstract

Naematolin is a biologically active sesquiterpene produced by *Hypholoma* species. Low titres and complex structure constrain the exploitation of this secondary metabolite. Here, we de novo sequenced the *H. fasciculare* genome to identify a candidate biosynthetic gene cluster for production of naematolin. Using *Aspergillus oryzae* as a heterologous host for gene expression, the activity of several sesquiterpene synthases were investigated, highlighting one atypical sesquiterpene synthase apparently capable of catalysing the 1,11 and subsequent 2,10 ring closures, which primes the synthesis of the distinctive structure of caryophyllene derivatives. Co-expression of the cyclase with an FAD oxidase adjacent within the gene cluster generated four oxidised caryophyllene-based sesquiterpenes: 5*β*,6*α*,8*β*-trihydroxycariolan, 5*β*,8*β*-dihydroxycariolan along with two previously unknown caryophyllene derivatives **2** and **3**. This represents the first steps towards heterologous production of such basidiomycete-derived caryophyllene-based sesquiterpenes, opening a venue for potential novel antimicrobials via combinatorial biosynthesis.

**Electronic supplementary material:**

The online version of this article (10.1007/s12033-019-00199-x) contains supplementary material, which is available to authorised users.

## Introduction

With the evolving resistance to almost all existing antibiotics, there is an urgent need for new classes of antibiotics that are naturally produced [1].

Lately, new classes of antibiotics have emerged based on terpene-based structures isolated from Basidiomycetes [2], but to date only a small number have been analysed in detail. There is therefore an urgent need to identify terpene pathways representing antimicrobial compounds in other basidiomycetes, to further add to potential useful compounds, and backbones for further chemical modification, leading to potential applications as clinical antibiotics.

Members of the basidiomycete genus *Hypholoma* produce structurally diverse terpenes, including sesquiterpenes [2]. Naematolin is a bicyclic sesquiterpene thought to be derived from the caryophyllene scaffold of the 1,11 and 2,10 carbon cyclisation of farnesyl pyrophosphate. This metabolite was first reported in *H. fasciculare* by Ito and co-workers [3] where its chemical structure was established based on spectroscopic and limited NMR data [4, 5]. Early biological investigations uncovered the antitumor and the antiviral properties of naematolin [6], but its production was in low titre and its complex structure precluded chemical synthesis, so the compound has remained comparatively unexploited.

Mining of this potential drug by genetic approaches has been limited in the past, due to the absence of efficient manipulation tools. However, the paradigm shift in genome sequencing projects provides a unique opportunity to re-discover naematolin isomers using bioinformatic tools. Sequence alignment is a useful tool in predicting gene function of core enzymes based on their conserved motif similarities. However, the use of such tool in linking the mature natural product to such genes is still challenging, as many synthase genes have been assigned to several precursors of structurally diverse chemicals [2]. This limitation triggered the need for genetic manipulation techniques to experimentally link predicted biosynthetic gene clusters to their chemical products. Two strategies of genetic engineering, featuring gene disruption and heterologous expression, were mainly utilised to underpin biosynthetic gene cluster in fungi [7], among which, heterologous expression proved to be the best option for gene function characterisation in basidiomycetes. When the entire pathway of a natural product of interest is considered, ascomycete hosts are more often selected, with *Aspergillus oryzae* being the most commonly used species [7]. Construction of a flexible genetic engineering platform enables yield and chemical structure optimisation of compound of interest and potential discovery of novel bioactive molecules. We have recently had success in analysis of other basidiomycete-derived terpenes such as the diterpene pleuromutilin from *Clitopilus passeckerianus* [8, 9] where we have not only linked the genetic pathway to the chemical synthesis, but have also used expression in a heterologous host to allow pathway manipulation and analysis. Pleuromutilin derivatives are now reaching the clinical market as a new class of antibiotics [8, 9].

Using similar techniques, we now report the isolation of a candidate gene cluster for naematolin, including heterologous expression of the first two biosynthetic genes (caryophyllene-like sesquiterpene cyclase and FADox tailoring gene), paving the way for further combinatorial biosynthesis of naematolin-based isomers, potentially leading to the generation of new antibiotic classes.

## Experimental Section

### Chemical Materials and Microbial Culture Conditions

Reagents and chemical standards were supplied from Fluka, Difco, Fisher, BDH, Sigma-Aldrich. Organic solvent used for LC–MS, HPLC and HRMS supplied from Fisher Scientific. Bacteria (*Escherichia coli* strains DH5α and ccdBS, *B. subtilis*, *K. pneumoniae*, *P. aeruginosa* and *S. aureus*) were maintained on Luria–Bertani Agar medium (10 g/L of NaCl, 10 g/L of tryptone, 5 g/L of yeast extract, 15 g/L of agar, pH 7). *H. fasciculare* and *H. sublateritium* were maintained on Potato Dextrose Agar medium (potato dextrose broth 24 g/L, agar 15 g/L) at 25 °C, and *A. oryzae* NSAR1 was maintained on supplemented Malt Extract Agar medium (MEA^+4^: malt extract 15 g/L, methionine 1.5 g/L, ammonium sulphate 2 g/L, arginine 1.5 g/L, adenine 0.1 g/L, agar 15 g/L) at 28 °C.

### Genome Mining and Computational Analysis

For sequence library preparation and identification of secondary metabolites enzymes, a large scale of *H. fasciculare* gDNA was isolated using the previously described method by [10]. The DNA is quantified and qualified using Nanodrop N1000, of which 500 ng is used to prepare a library of size 702 bp using Illumina Truseq Nano DNA kit. The produced data were analysed using HiSeq Control Software 2.258 and assembled using short read assembler de novo metagenomic IDBA-UD.

*Hypholoma fasciculare* genome was further mined and manually inspected using antiSMASH and blast search using previously characterised enzymes, involving *Coprinopsis cinerea* sesquiterpene synthases with different carbon cyclisation patterns [11, 12]. Artemis Comparison Tool (ACT) was also used to compare selected genomic regions of *H. fasciculare* with its related species *H. sublateritium*. Gene annotation and phylogenetic reconstructions were performed using published methods [13, 14].

### Sample Preparation for GC–MS Analysis

Spores of *A. oryzae* were inoculated into 100 mL CMP (Czapek-dox broth 35 g/L, maltose 20 g/L, peptone 10 g/L) in a 250-mL flask and incubated for 7 days with shaking. When grown, 30 mL of Hexane was added to each flask, homogenised, mixed for 20 min at room temperature and then filtered. The organic phase of this mixture was collected and dried over anhydrous MgSO_4_ to give the crude extract, of which 1 mL was then analysed by GC–MS analysis (see Supplementary information).

### HPLC–MS Analysis

For analysis by HPLC, cultures were grown as above but extracted into ethyl acetate. After filtration and removal of residual water, the solvent was evaporated and crude extract resuspended in acetonitrile at 50 mg/mL. Crude purification of naematolin was performed using column chromatography with silica gel (Sephadex LH-20, MCI gel CHP 2OP), eluting with methanol.

The partially purified fractions from flash columns were further purified by preparative reverse-phase HPLC–MS, collecting novel compounds on the basis of UV/ELSD and Rt using a Waters mass-directed collector, connected to a Waters 2767 automated sample injector, equipped with Waters 2545 pump, and a Phenomenex LUNA C18, 2.6 *μ,* 100 Å, 4.6 × 100 mm column and a Phenomenex Security Guard precolumn Luna C_5_ 300 Å.

### NMR and HRMS Analysis

All purified metabolites were characterised using Agilent VNMRS500 (500 MHz) NMR spectrometer. 1 mg/mL of each sample was dissolved in either methanol-d4 (CD_3_OD) or chloroform-d4 (CDCl_3_). Chemical shifts were recorded in parts per million unit (ppm) and the coupling constant (J) recorded in Hz. All chemical shifts are reported relative to the solvent. ^1^H-NMR CHCl_3_ = 7.24 singlet or CH_3_OH = 4.78 singlet; ^13^C-NMR shifts were recorded relative to ^13^C resonance of chloroform = 77.00 triplet, or methanol = 49.00 quintet. Compound ionisation patterns were analysed using a Bruker Daltonics microTOF focus with either positive or negative ESI.

### Heterologous Expression of *H. fasciculare* genes in *A. oryzae*

For RNA extraction of *H. fasciculare,* the TRIzol method [15] was modified (see RNA extraction supplementary information). Full-length cDNA for each desired gene was obtained by RT-PCR and recombined into *A. oryzae* expression vectors by yeast-based recombination methods [16]. Appropriately constructed plasmids were transformed into *A. oryzae* [17]. Six independent PCR-positive transformants were analysed chemically for each combination of plasmids.

## Results

### Naematolin Re-characterisation

*Hypholoma fasciculare* and *H. sublateritium* FD-344 SS-4 were both cultured in 100 mL YMG liquid media [18] and were evaluated for naematolin production by LCMS. A major product with Rt of 12.25 min corresponded with naematolin. Purification and HRMS indicated a compound with m/z 331.1522 (consistent with C_17_H_24_NaO_5_). IR and NMR (Figs. S3 to S9 and Table S1 Supplementary information) were also in complete agreement with the literature [3–5], confirming naematolin production by both species. The antibacterial properties of naematolin were confirmed by disc diffusion assays, showing some limited activity against *Bacillus subtilis* and *Staphylococcus aureus*, but no activity against *Pseudomonas aeruginosa* or *Klebsiella pneumoniae* (Table S2).

### Genome Sequencing and Analysis

The genome of *H. fasciculare* was sequenced using the short read assembler de novo metagenomic IDBA-UD. This assembly afforded a draft genome of 58.84 Mbp with contig N50 of 49,633 (see Table S4 supplementary information). To assess whether there was synteny between *H. fasciculare* and the publicly available genome of *H. sublateritium*, the genomic loci for two housekeeping genes (*gpd* and *β*-*tubulin*) were identified, and open reading frames ± 20 kb of the locus were aligned using Artemis. Comparisons of all genes within the selected genomic regions showed high sequence similarity (> 80%) and were in the same orientation highlighting considerable synteny between these species (Figs. S10 and S11 Supplementary information).

**Fig. 1:**
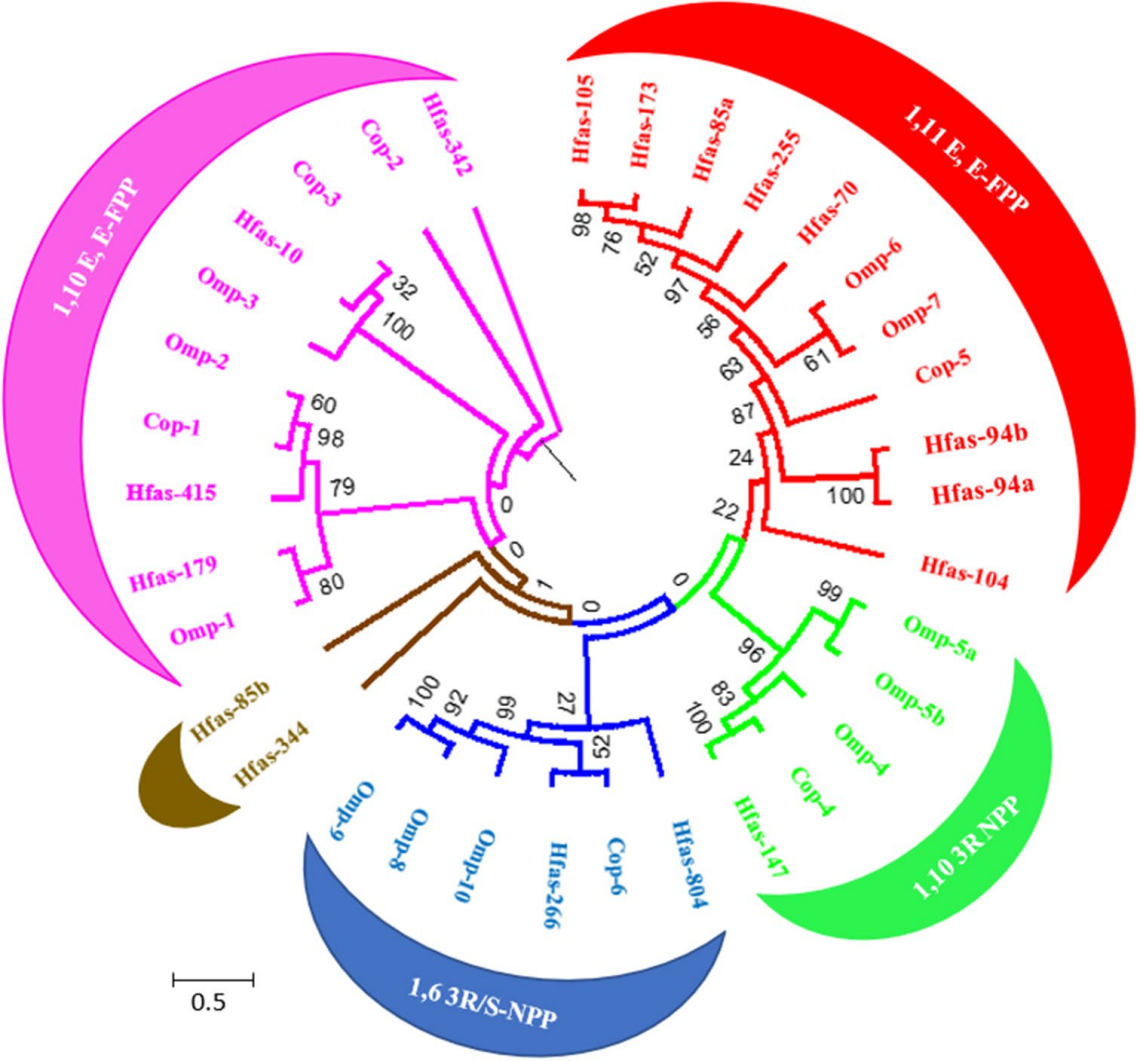
Maximum likelihood tree of *H. fasciculare* (Hfas) terpene synthases proteins. The clades compromising Hfas putative terpene synthases and sesquiterpene synthase from *C. cinerea* (Cop) and *O. olearius* (Omp), based on sesquiterpene synthase homologs and their initial cyclisation reaction. The Cop and Omp sequences were obtained from JGI. Contigs or scaffold numbers are shown adjacent to species abbreviations. *1,11 E, E-FPP* 1, 11 E. E Farnesyl diphosphate. *1, 10 3R NPP* 3R-Nerolidyl diphosphate

Potential secondary metabolite gene clusters were identified in both fungi by a combination of AntiSmash [19] along with BLAST searches for specific classes of enzyme. Together this identified seventeen putative sesquiterpene synthases (SQS) in *H. fasciculare*. A maximum likelihood phylogeny comparison with recently characterised sesquiterpene synthase from two basidiomycetes (*Omphalotus olearius* and *Coprinopsis cinerea*) [20, 21] placed most *H. fasciculare* SQS in four different clades, indicating their likely activity in terms of mode of terpene skeleton cyclisation (Fig. 1). Eight of these proteins were predicted as 1,11 carbon cyclisation enzymes, of which two (Hfas-94a and Hfas-94b) were 87% identical to omp-6 and omp-7, previously characterised as protoilludene synthases [21]. Of the remaining SQS enzymes, Hfas-147 clustered with the 1,10 ring closure of 3R NPP, Hfas-804 and Hfas-266 were placed within the clade responsible for the 1,6 ring closure of 3R/S-NPP, and Hfas-179, Hfas-415, Hfas-10, Hfas-342 were all present in the clade catalysing the 1,10 ring closure of E,E-FPP. However, two (Hfas-344 and Hfas85b) were not placed within the usual clades, perhaps indicative of different types of sesquiterpene cyclisation.

### Chemical Analysis of Transgenic *A. oryzae*

**Fig. 2:**
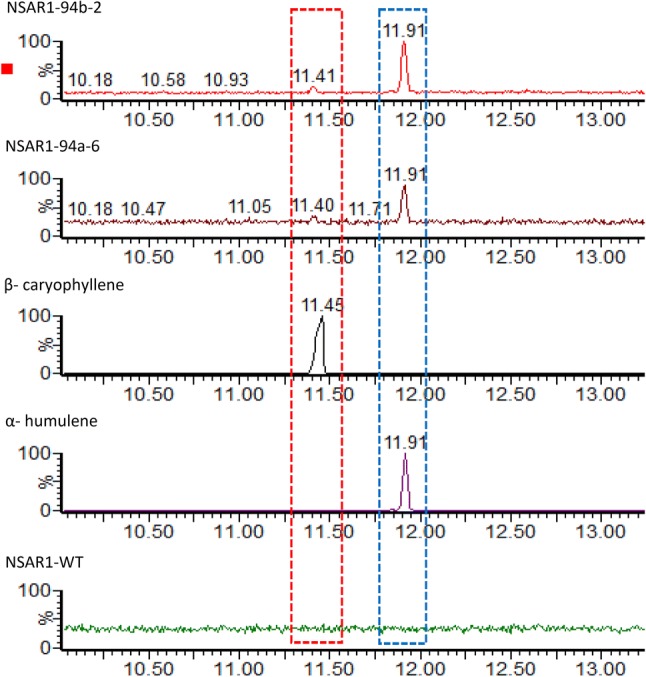
GC–MS analysis of crude extracts from wild-type and SQS transformants of *A. oryzae*. **a** NSAR1-Hf94b, **b** NSAR1-Hf94a, **c***β*-caryophyllene chemical standard, **d** α-humulene chemical standard and **e** NSAR1-WT

Each of the SQS predicted to deliver a 1,11 cyclisation pattern (Hfas94a, Hfas94b and Hfas255) was individually cloned into an expression vector for *A. oryzae* and six independent transformants assessed by GC–MS for production of novel compounds, or enhanced titres of existing compounds. Transformants with Hfas94a and Hfas94b both yielded α-humulene at Rt 11.91 as major compound along with minor traces consistent with *β*-caryophyllene at Rt 11.41 (Fig. 2).

**Fig. 3:**
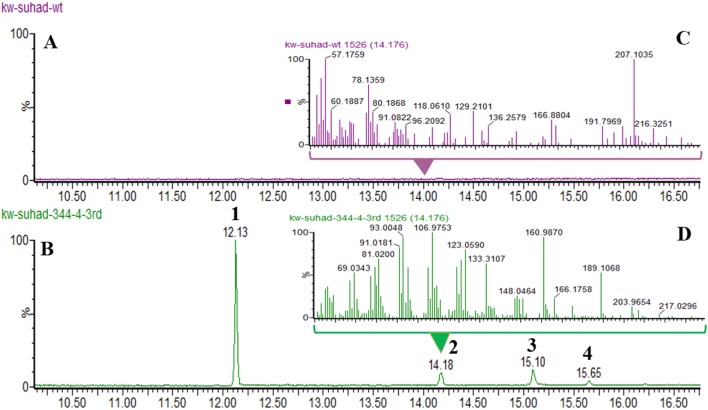
GC–MS analysis of crude extract from wild-type and Hfas344 transformants. **a**, **b** LC–MS profile of NSAR1-WT and NSAR-344 transformants-4, respectively. **c**, **d** Mass spectrum fragmentation (ES+) of NSAR1-WT and NSAR-344 transformants-4, respectively, measured at 14.18 min, indicating a coeluting novel product in the transformant

Expression of Hfas255 delivered no new products. Given that expression of the candidate genes from the 1,11 SQS clade failed to deliver efficient caryophyllene production, the atypical SQS Hfas-344 was also transformed into *A. oryzae*. This SQS still contains the expected D(D/E)xxD and NSE domains characteristic of SQS, but had little other sequence similarity to the canonical SQS from *H. fasciculare*. Successful expression of Hfas344 in *A. oryzae* led to accumulation of four new metabolite peaks: a major product at 12.13 min and minor products at 14.18, 15.10 and 15.65 min. When compared with the NIST MS spectra database, peaks 1 and 2 were both consistent with caryophyllene isomers (although not the Rt of β-caryophyllene), product 3 was likely an oxidised sesquiterpene, whilst peak 4 showed no significant matches (Figs. S12, S13, S14 and S15 Supplementary information (Fig. 3).

### Heterologous Expression of Additional Naematolin Biosynthesis Genes

**Fig. 4:**
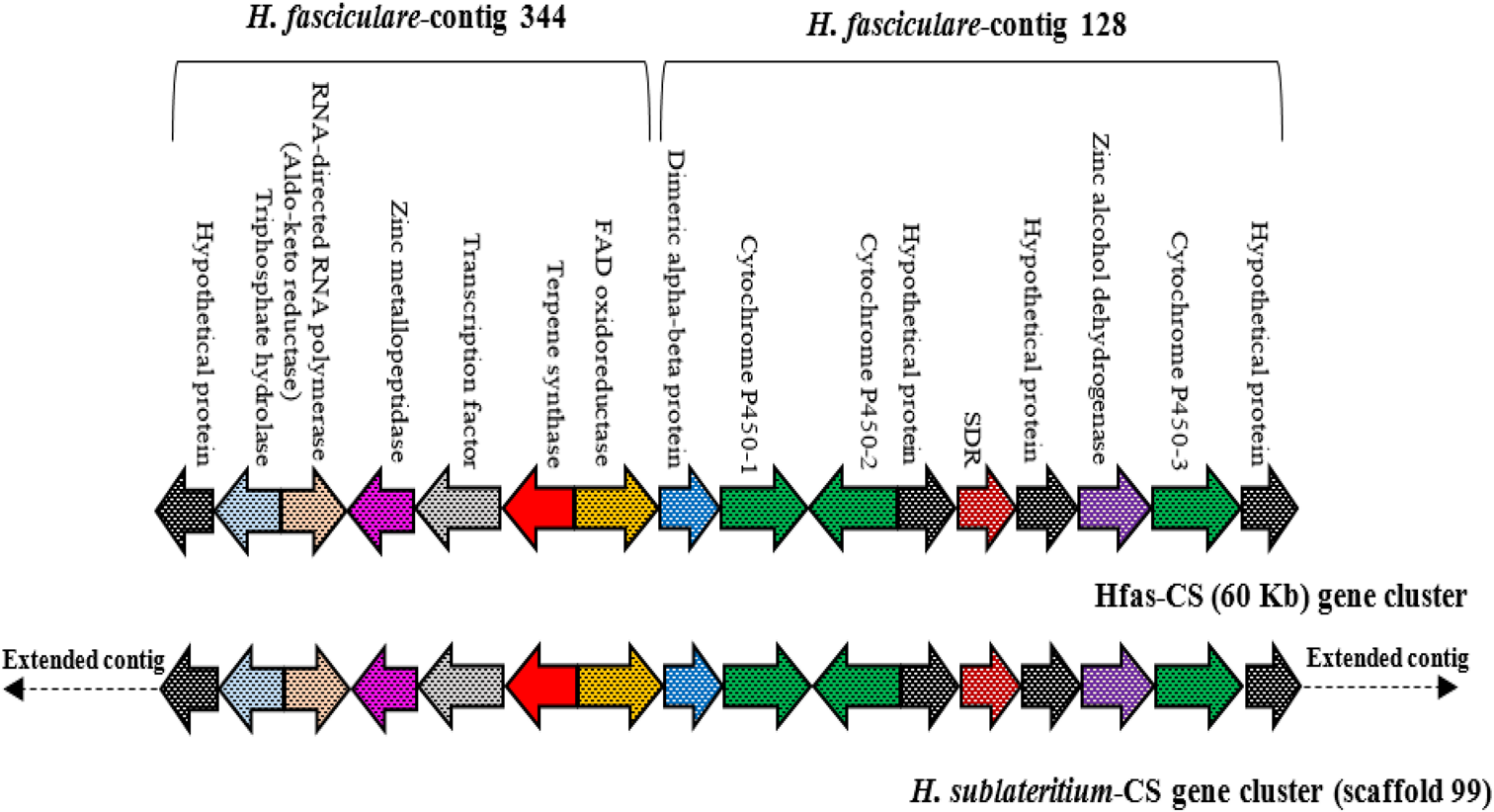
Comparison of the predicted gene cluster of the HFas344 locus for *H. fasciculare* and *H. sublateritium*

The candidate naematolin SQS Hfas-344 genome locus was aligned with the homologous cluster of *H. sublateritium* based around SQS Hsub99. The Hsub99 contig was larger, extending beyond Hfas344 and allowed identification of the presumed adjacent region from *H fasciculare*, contig Hfas128 (Fig. 4). This revealed a number of candidate tailoring enzymes, including an FAD oxidoreductase (FADox), aldoketoreductase, zinc-dependent carboxy-peptidase, zinc alcohol dehydrogenase and two cytochrome P450 oxidoreductases (Fig. 4 and Table S5). Whilst the cDNA of some of these genes was difficult to generate, the FADox protein was predicted, so this gene was cloned into an expression plasmid along with the Hfas344 SQS.

Following transformation of *A. oryzae* with the dual expression plasmid, the chemical profile of transformants was investigated by LC–MS of fungal extracts. The caryophyllene-isomer peak disappeared, and six new peaks were observed, four of which were purified by flash column chromatography and preparative HPLC (Fig. 5). Exact masses, IR and 2D NMR analysis were performed to elucidate their structures (Figs. S16 to S47 and Tables S6 and S7). All of these molecules appear to be based on a caryophyllene-like core, so were consistent with the proposed identification of the cluster as being responsible for naematolin biosynthesis. Two compounds were found to be known having previously been reported from the Birch tree *Betula pendula* [22, 23], namely (5*β*,6*α*,8*β*-trihydroxycariolan [1] and 5*β*, 8*β*-dihydroxycariolan [4, 5] (Fig. 6a, d, respectively) but are a new report from fungi, whilst 2 and 3 appear to be novel.

**Fig. 5:**
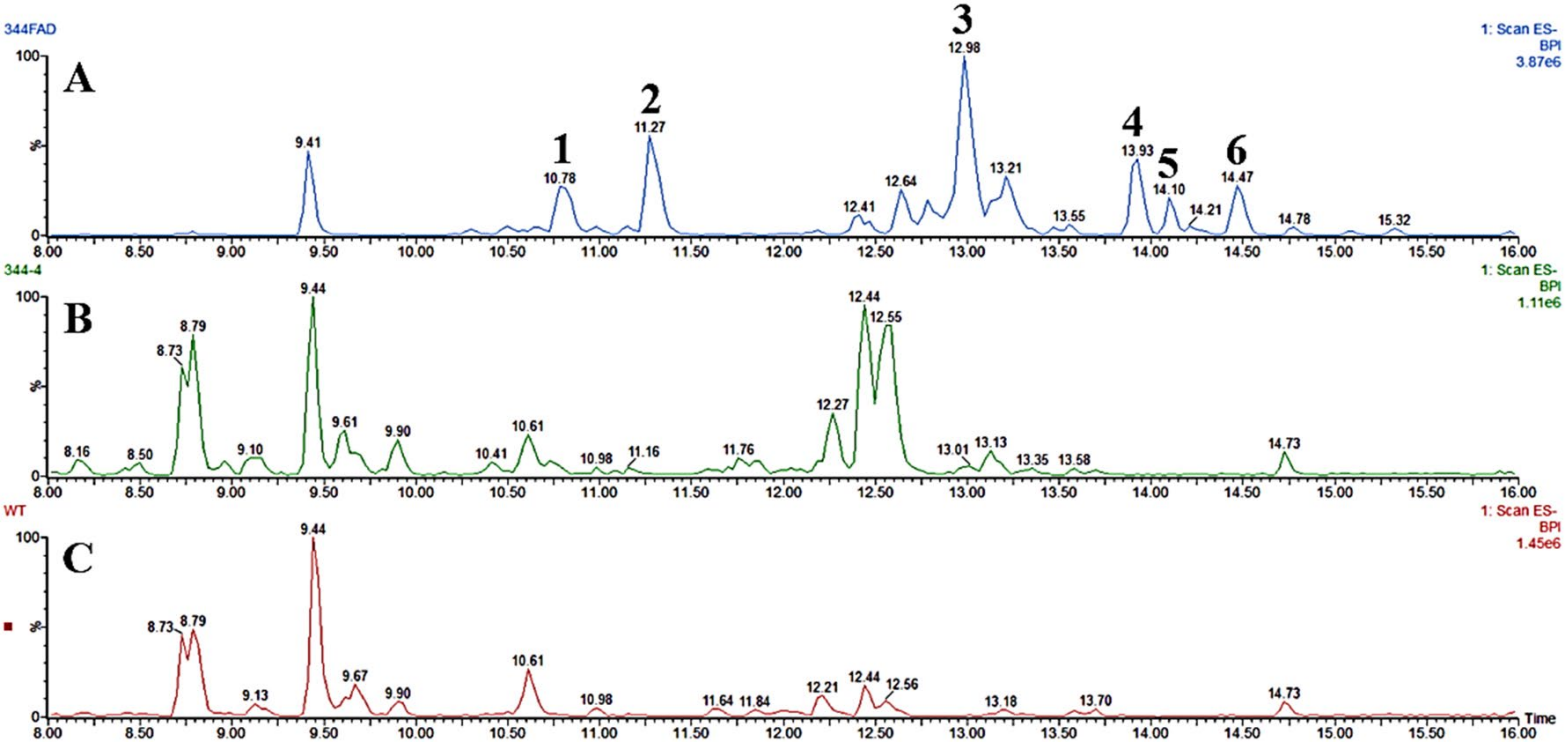
LC–MS profile from 7.5–16.50 min of **a** NSAR1 + Hfas344SQS + FADox; **b** NSAR1 + Hfas344 SQS; **c** NSAR1. In total, six new metabolites were detected within this dual expression transformant

**Fig. 6:**
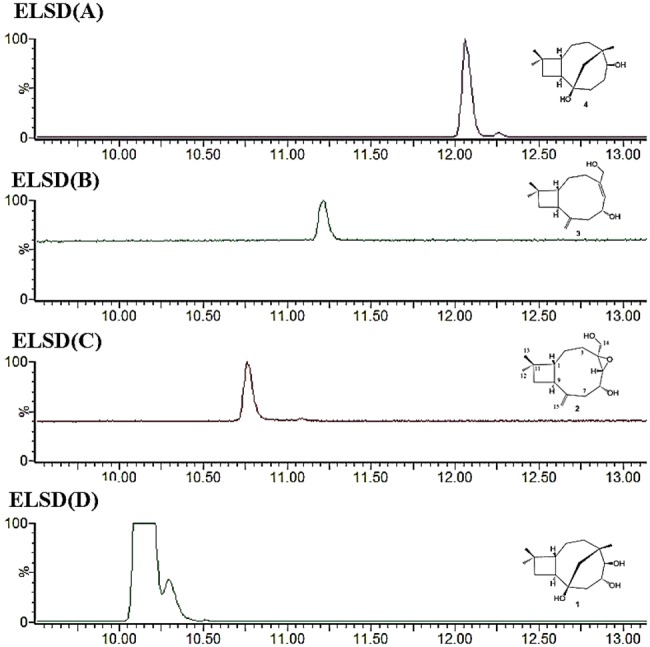
HPLC (ELSD) of **a** compound **4**, **b** compound **3**, **c** compound **2** and **d** compound **1**

Compound 2 (Fig. 6c) was isolated as a colourless oil; $\left[ \alpha \right]_{\text{D}}^{25}$ − 23.6° (c 0.1, CHCl_3_); IR *ν*_max_ 3350, 2926, 1448, 1034 cm^−1^; δ_H_ (500 MHz, CDCl_3_) 0.83 (1H, m, H-3a), 0.91 (3H, s, H-12), 1.20 (3H, s, H-13), 1.54 (2H, m, H-2), 1.66 (1H, m, H-10a), 1.76 (1H, m, H-10b), 1.93 (1H, m, H-7a), 2.09 (1H, m, H-1), 2.51 (1H, m, H-3b), 2.86 (1H, d, J = 10.0, H-5), 2.94 (1H, m, H-7b), 3.08 (1H, m, H-9), 3.50 (1H, d, J = 15.0 Hz, H-14a), 3.77 (1H, d, J = 15.0 Hz, H-14b), 3.80 (1H, m, H-6), 5.02 (1H, br s, H-15a), 5.03 (1H, br s, H-15b); δC (125 MHz, CDCl3) 21.0 (C-2), 25.2 (C-12), 29.7 (C-13), 33.3 (C-11), 35.7 (C-3), 38.0 (C-10), 42.2 (C-9), 45.4 (C-7), 52.9 (C-1), 62.9 (C-14), 63.4 (C-4), 66.4 (C-5), 69.3 (C-6), 116.1 (C-15), 144.7 (C-8); HRESIMS m/z 275.1625 [M+Na]^+^ (calcd for C_15_H_24_O_3_Na, 275.1623).

Compound 3 (Fig. 6b) was also a colourless oil; $\left[ \alpha \right]_{\text{D}}^{25}$ − 38.4° (*c* 0.15, CHCl_3_); IR *ν*_max_ 3325, 2972, 1379, 1046 cm^−1^; δ_H_ (500 MHz, CDCl_3_) 0.90 (3H, s, H-12), 1.21 (3H, s, H-13), 1.48 (2H, m, H-2), 1.50 (1H, m, H-10a), 1.70 (1H, m, H-10b), 1.78 (1H, m, H-7a), 1.80 (1H, m, H-3a), 2.11 (1H, m, H-1), 2.52 (1H, m, H-3b), 2.87 (1H, m, H-7b), 2.93 (1H, m, H-9), 3.88 (1H, d, *J* = 12.5 Hz, H-14a), 4.18 (1H, d, *J* = 12.5 Hz, H-14b), 4.62 (1H, m, H-6), 4.80 (1H, br s, H-15a), 4.81 (1H, br s, H-15b), 5.20 (1H, d, *J* = 10.5 Hz, H-5); δ_C_ (125 MHz, CDCl_3_) 25.6 (C-12), 26.0 (C-2), 29.6 (C-13), 33.3 (C-11), 36.3 (C-3), 37.7 (C-10), 42.9 (C-9), 48.3(C-7), 52.3 (C-1), 61.6 (C-14), 68.7 (C-6), 113.7 (C-15), 128.2 (C-5), 139.7 (C-4), 148.0 (C-8); HRESIMS *m/z* 259.1675 [M+Na]^+^ (calcd for C_15_H_24_O_2_Na, 259.1674).

### Bioactivity Test for Isolated Compounds

Compounds 1–4 from the transgenic *A. oryzae* were assayed by disc diffusion for antibiotic properties against a panel of microbes. Compounds 1–4 all showed some weak activity against *B. subtilis*, although not as much as naematolin and none had any effect on the other bacteria in the test panel (Tables 1 and 2).

**Table 1:** Antimicrobial activity of four isolated caryophyllene isomers tested against 4 bacterial species: *Staphylococcus aureus*, *Pseudomonas aeruginosa*, *Klebsiella pneumoniae* and *B. subtilis*

Microbe	Clearing zone (mm) disc diameter 5 mm																	
	100 μg/disc Kanamycin +ve control	100 μL/disc 98% methanol −ve control	Compound 1				Compound 2				Compound 3				Compound 4			
			100 μg/disc	200 μg/disc	500 μg/disc	1000 μg/disc	100 μg/disc	200 μg/disc	500 μg/disc	1000 μg/disc	100 μg/disc	200 μg/disc	500 μg/disc	1000 μg/disc	100 μg/disc	200 μg/disc	500 μg/disc	1000 μg/disc
*B. subtilis*	12	–	–	–	–	2	–	–	8	12	–	–	6	8	–	–	–	4
*S. aureus*	11	–	–	–	–	–	–	–	–	–	–	–	–	–	–	–	–	–
*P. aeruginosa*	4	–	–	–	–	–	–	–	–	–	–	–	–	–	–	–	–	–
*K. pneumoniae*	8	–	–	–	–	–	–	–	–	–	–	–	–	–	–	–	–	–

**Table 2:** Antimicrobial activity of naematolin tested 4 bacterial species: *Staphylococcus aureus*, *Pseudomonas aeruginosa*, *Klebsiella pneumoniae* and *B. subtilis*

Microorganism	Clearing zone (nn) disc diameter 5 mm					
	100 μg/disc kanamycin (+ve control)	100 μL of 98% methanol (−ve control)	50 μg/disc naematolin	100 μg/disc naematolin	200 μg/disc naematolin	500 μg/disc naematolin
*B. subtilis*	12	–	6	8	10	14
*S. aureus*	11	–	–	6	9	13
*P. aeruginosa*	4	–	–	–	–	–
*K. pneumoniae*	8	–	–	–	–	–

### Accession Numbers

The verified sequences of Hfas94a, Hfas94b and Hfas344 can be found on NCBI under MK287936, MK287937 and MK287938, respectively.

## Discussion

To date, no caryophyllene synthase has been identified from basidiomycetes, revealing such genes encoding novel biochemical function would therefore provide an important tool to develop potential new antimicrobial compounds.

Naematolin is a modified form of a caryophyllene isomer. To date, there are no reported caryophyllene synthases from basidiomycete fungi, precluding the direct identification of such genes in *H. fasciculare*. Therefore, all the terpene synthases predicted within *H. fasciculare* genome were investigated, first by whether there was a corresponding homologue in the *H. sublateritium* genome (as both produce naematolin), and if so, were there enough candidate tailoring genes also present at the same locus to deliver production of the mature compound. Our heterologous expression of Hfas94a and Hfas94b confirmed that both enzymes produce humulene as their major product as was predicted given that Hfas94a and 94b demonstrated high sequence similarity with *O. olearius* enzymes Omp-6 and Omp-7 that produce protoilludane, highlighting the promiscuous feature of sesquiterpene synthases [21]. This ruled out the SQS from the 1,11-cyclisation clade from being responsible for caryophyllene production. Hfas344 is an atypical SQS that does not cluster into the conventional clades, so its cyclisation pattern could not be predicted; however, its expression in *A. oryzae* generated several products, the major most likely being a caryophyllene isomer, further demonstrating the limitation of bioinformatic predictions of gene function.

In connection with conserved motifs, DDxxD and NSE are the most common ones of SQS enzymes apparently responsible for their catalytic activity; however, advanced biochemical investigations on these motifs suggested the presence of more specific catalytic motifs in such enzymes [22–26]. Sequence alignment of our SQS with chemically characterised SQS supported the presence of other conserved residues in Hfas344 SQS (Fig. S47 supplementary information). Biochemical analysis of such residues, however, is a prerequisite to further confirm their role in the catalytic activity of such enzymes. Like us, Cox and co-workers could not draw a definite conclusion of motifs responsible for their humulene synthase (AsR6) catalytic activity [27].

Following our successful expression of Hfas344 in *A. oryzae*, we then co transformed it with its adjacent FADox biosynthetic gene, allowing the production of two novel caryophyllene oxides (compounds 2 and 3), along with two previously known oxygenated caryophyllene isomers (compounds 1 and 4). The presence of more than one hydroxyl group in the produced caryophyllene isomers suggests that FADox is multifunctional [28]. Such multifunctionality of tailoring enzymes has been demonstrated in the biosynthesis of both aphidicolin and ophiobolin, where one cytochrome P450 oxidoreductase catalyses two [29] and four oxidations [30], respectively. The possibility remains however that some of these additional oxidations observed in the production of 1–4 are performed by the host rather than as a result of the transgenes, but if this is the case, they are only possible following the prior action of the FADox, as they are not observed when the Hfas344 SQS alone has been expressed.

## Conclusion

In this work, we aimed to assign the biosynthetic gene cluster of naematolin. Naematolin is a bioactive bicyclic sesquiterpene that naturally initiates from caryophyllene core in *Hypholoma* species. Its antiviral, antioxidant and antimicrobial activity is well documented. However, its development as potential drug has been hindered by its low titre and structure complexity. To scale up production and further modify its chemical structure for the development of novel bioactive molecules, its biosynthetic gene cluster was a prerequisite. Our start point was sequencing the whole genome of the native producer *Hypholoma* and predict all potential terpene synthases. This was followed by sequence analysis and heterologous expression of putative SQS with their related tailoring genes in *A. oryzae*, allowing the production of caryophyllene and four analogous derivatives of which two have shown novel chemical structure. Caryophyllene itself has many medical properties, including insecticidal, apoptosis stimulator, antileishmanial and antifungal activities [31–36], indicating its biosynthetic gene could help in the development of novel caryophyllene derivatives, especially that its oxygenated forms have shown antimicrobial activity against *B. subtilis* throughout this research.

This is the first report of caryophyllene synthase being identified from basidiomycetes and of a FAD tailoring gene involving in two oxidation reactions. Revealing such genes involved in sesquiterpene antimicrobials will therefore provide an important tool to enhance effects of nature-based drugs.

## Electronic supplementary material

Below is the link to the electronic supplementary material.

Supplementary material 1 (DOCX 92244 kb)

## References

[1] Butler MS, Blaskovich MA, Cooper MA (2013). Antibiotics in the clinical pipeline in 2013. Journal of Antibiotics.

[2] Quin MB, Flynn CM, Schmidt-Dannert C (2014). Traversing the fungal terpenome. Natural Products Reports.

[3] Ito Y, Kurita H, Yamaguchi T, Sato M, Okuda T (1967). Naematolin, a new biologically active substance produced by *Naematoloma fasciculare* (Fr.) Karst. Chemical & Pharmaceutical Bulletin.

[4] Backens S, Steffan B, Steglich W, Zechlin L, Anke T (1984). Antibiotika aus basidiomyceten, XIX naematolin und naematolon, zwei caryophyllan-derivate aus kulturen von Hypholoma-Arten (Agaricales). Liebigs Annalen der Chemie.

[5] de Mattos-Shipley KMJ, Ford KL, Alberti F, Banks AM, Bailey AM, Foster GD (2016). The good, the bad and the tasty: The many roles of mushrooms. Studies in Mycology.

[6] Abraham WR (2001). Bioactive sesquiterpenes produced by fungi are they useful for humans as well. Current Medicinal Chemistry.

[7] Greco C, Pfannenstiel BT, Liu JC, Keller NP (2019). *Depsipeptide Aspergillicins* revealed by chromatin reader protein deletion. ACS Chemical Biology.

[8] Bailey AM, Alberti F, Kilaru S, Collins CM, de Mattos-Shipley K, Hartley AJ, Hayes P, Griffin A, Lazarus CM, Cox RJ, Willis CL (2016). Identification and manipulation of the pleuromutilin gene cluster from *Clitopilus passeckerianus* for increased rapid antibiotic production. Scientific Reports.

[9] Alberti F, Khairudin K, Rodriguez-Venegas E, Davies JA, Hayes PM, Willis CL, Bailey AM, Foster GD (2018). Heterologous expression reveals the biosynthesis of the antibiotic pleuromutilin and generates bioactive semi-synthetic derivatives. Nature Communications.

[10] Möller EM, Bahnweg G, Sandermann H, Geiger HH (1992). A simple and efficient protocol for isolation of high molecular weight DNA from filamentous fungi, fruit bodies, and infected plant tissues. Nucleic Acids Research.

[11] Yoshikuni Y, Ferrin TE, Keasling JD (2006). Designed divergent evolution of enzyme function. Nature.

[12] Yoshikuni Y, Martin VJ, Ferrin TE, Keasling JD (2006). Engineering cotton ()-delta-cadinene synthase to an altered function: Germa creneD-4-ol synthase. Chemistry & Biology.

[13] Thompson JD, Gibson TJ, Plewniak F, Jeanmougin F, Higgins DG (1997). The CLUSTAL_X windows interface: Flexible strategies for multiple sequence alignment aided by quality analysis tools. Nucleic Acids Research.

[14] Kumar S, Stecher G, Tamura K (2016). MEGA7: Molecular evolutionary genetics analysis version 7.0 for bigger datasets. Molecular Biology and Evolution.

[15] Simms D, Cizdziel PE, Chomczynski P (1993). TRIzol: A new reagent for optimal single-step isolation of RNA. Focus.

[16] Lazarus CM, Williams K, Bailey AM (2014). Reconstructing fungal natural product biosynthetic pathways. Natural Product Reports.

[17] Halo LM, Heneghan MN, Yakasai A, Song Z, Williams K, Bailey AM, Cox RJ, Lazarus CM, Simpson TJ (2008). Late stage oxidations during the biosynthesis of the 2-pyridone tenellin in the entomopathogenic fungus *Beauveria bassiana*. Journal of the American Chemical Society.

[18] Al-Salihi SA, Scott TA, Bailey AM, Foster GD (2017). Improved vectors for *Agrobacterium* mediated genetic manipulation of *Hypholoma* spp. and other homobasidiomycetes. Journal of Microbiol Methods.

[19] Blin K, Wolf T, Chevrette MG, Lu X, Schwalen CJ, Kautsar SA, Duran S, Hernando G, de los Santos EL, Kim HU, Nave M (2017). antiSMASH 4.0— improvements in chemistry prediction and gene cluster boundary identification. Nucleic Acids Research.

[20] Agger S, Lopez-Gallego F, Schmidt-Dannert C (2009). Diversity of sesquiterpene synthases in the basidiomycete *Coprinus cinereus*. Molecular Microbiology.

[21] Wawrzyn GT, Quin MB, Choudhary S, López-Gallego F, Schmidt-Dannert C (2012). Draft genome of *Omphalotus olearius* provides a predictive framework for sesquiterpenoid natural product biosynthesis in Basidiomycota. Chemistry & Biology.

[22] Vedernikov DN, Roshchin VI (2012). Extractive compounds of Betulaceae family Birch buds (Betula pendula Roth.): IV. Composition of sesquiterpene diols, triols and flavanoids. Russian Journal of Bioorganic Chemistry.

[23] Koellner TG, O’Maille PE, Gatto N, Boland W, Gershenzon J, Degenhardt J (2006). Two pockets in the active site of maize sesquiterpene synthase TPS4 carry out sequential parts of the reaction scheme resulting in multiple products. Archives of Biochemistry and Biophysics.

[24] López-Gallego F, Wawrzyn G, Schmidt-Dannert C (2010). Selectivity of fungal sesquiterpene synthases: Role of the active site’s H-1α loop in catalysis. Applied and Environmental Microbiology.

[25] Greenhagen BT, O’Maille PE, Noel JP, Chappell J (2006). Identifying and manipulating structural determinates linking catalytic specificities in terpene synthases. Proceedings of the National academy of Sciences of the United States of America.

[26] O’Maille PE, Malone A, Dellas N, Andes-Hess B, Smentek L, Sheehan I, Greenhagen BT, Chappell J, Manning G, Noel JP (2008). Quantitative exploration of the catalytic landscape separating divergent plant sesquiterpene synthases. Nature Chemical Biology.

[27] Schor R, Schotte C, Wibberg D, Kalinowski J, Cox RJ (2018). Three previously unrecognised classes of biosynthetic enzymes revealed during the production of xenovulene A. Nature Communications.

[28] Tahallah N, van den Heuvel RH, van den Berg WA, Maier CS, van Berkel WJ, Heck AJ (2002). Cofactor-dependent assembly of the flavoenzyme vanillyl-alcohol oxidase. Journal of Biological Chemistry.

[29] Hu J, Okawa H, Yamamoto K, Oyama K, Mitomi M, Anzai H (2011). Characterization of two cytochrome P450 monooxygenase genes of the pyripyropene biosynthetic gene cluster from *Penicillium coprobium*. Journal of Antibiotics.

[30] Narita K, Minami A, Ozaki T, Liu C, Kodama M, Oikawa H (2018). Total Biosynthesis of Antiangiogenic Agent (−)-Terpestacin by Artificial Reconstitution of the Biosynthetic Machinery in Aspergillus oryzae. Journal of Organic Chemistry.

[31] Van Alfen NK (2014). Encyclopedia of agriculture and food systems.

[32] Legault J, Pichette A (2007). Potentiating effect of β-caryophyllene on anticancer activity of α-humulene, isocaryophyllene and paclitaxel. Journal of Pharmacy and Pharmacology.

[33] Pant A, Saikia SK, Shukla V, Asthana J, Akhoon BA, Pandey R (2014). Beta-caryophyllene modulates expression of stress response genes and mediates longevity in *Caenorhabditis elegans*. Experimental Gerontology.

[34] Soares DC, Portella NA, Ramos MFDS, Siani AC, Saraiva EM (2013). Trans-β-caryophyllene: An effective antileishmanial compound found in commercial copaiba oil (Copaifera spp.). Evidence-Based Complementary and Alternative Medicine.

[35] Liu H, Yang G, Tang Y, Cao D, Qi T, Qi Y, Fan G (2013). Physicochemical characterization and pharmacokinetics evaluation of β-caryophyllene/β-cyclodextrin inclusion complex. International Journal of Pharmaceutics.

[36] Minerdi D, Bossi S, Gullino ML, Garibaldi A (2009). Volatile organic compounds: A potential direct long-distance mechanism for antagonistic action of *Fusarium oxysporum* strain MSA35. Environmental Microbiology.

