# Supplementary material for: The Biogenetic Origin of the Biologically Active Naematolin of Hypholoma Species Involves an Unusual Sesquiterpene Synthase
Source: Mol Biotechnol. 2019 Aug 7;61(10):754–62. doi: 10.1007/s12033-019-00199-x (PMC7019648; doi:10.1007/s12033-019-00199-x)
Supplement: Supplementary file 1 — Supplementary material 1 (DOCX 92244 kb) [file 12033_2019_199_MOESM1_ESM.docx]

**The biogenetic origin of the biologically active naematolin of *Hypholoma* species involves an unusual sesquiterpene synthase**

Suhad A.A. Al-Salihi^1,3*^, Trong Tuan Dao^2^, Katherine Williams^1^, Andy M. Bailey^1^, Gary D. Foster^1*^

1 School of Biological Sciences, University of Bristol, 24 Tyndall Avenue Bristol, BS8 1TQ, UK

2 School of Chemistry, University of Bristol, Cantock’s Close, Bristol, BS8 1TS, UK

3 Current address: Applied Sciences Department, University of Technology, Baghdad, Iraq.

^*^To whom correspondence should be addressed: Gary Foster [Gary.Foster@bristol.ac.uk](mailto:Gary.Foster@bristol.ac.uk) and Suhad Al-Salihi [suhadbiotechnology@gmail.com](mailto:suhadbiotechnology@gmail.com)

**Supplementary Content**

| **Methods**  **GC-MS sampling**  **RNA extraction**  ***A. oryzae* mutants**  **Production of dual-gene mutant strains**  **Figures and Tables**  **References**  Figure S1: LC-MS chromatogram (UV absorbance/retention time) of *H. fasciculare* (CGC culture). |  |
| --- | --- |
| Figure S2: High resolution mass (HRMS) of purified naematolin |  |
| Figure S3: Naematolin infrared (IR) spectrum |  |
| Figure S4: Naematolin predicted chemical structure |  |
| Figure S5: ^1^H NMR spectrum (CDCl_3_, 125 MHz) of naematolin |  |
| Figure S6: ^13^C NMR (500 MHz) spectrum (CDCl_3_, 125 MHz) of Naematolin |  |
| Figure S7: COSY (500 MHz) spectrum of naematolin |  |
| Figure S8: HSQC (500 MHz) spectrum of Naematolin |  |
| Figure S9: HMBC (500 MHz) spectrum of Naematolin |  |
| Table S1: Carbon and proton chemical shift position for naematolin of *H. sublateritium* |  |
| Figure S10: A comparison of the *gpd* gene region from *H. fasciculare* and the *gpd* gene region from *H. sublateritium* using Artemis Comparison Tool (ACT) |  |
| Figure S11: A comparison of the *β-tubulin* gene region from *H. fasciculare* and *H. sublateritium* using Artemis Comparison Tool (ACT). |  |
| Table S2: Genome assembly statistic of both *H. sublateritium and H. fasciculare.* |  |
| Figure S12: Shows mass spectrum comparison of NSAR1-Hfas344 transformant-4 of metabolite-1 eluted at 12.12. |  |
| Figure S13: Shows mass spectrum comparison of NSAR1-Hfas344 transformant-4 of metabolite-2 eluted at 14.18. |  |
| Figure S14: Shows mass spectrum comparison of NSAR1-Hfas344 transformant-4 of metabolite-3 eluted at 15.10. |  |
| Figure 15: Shows mass spectrum comparison of NSAR1-Hfas344 transformant-4 of metabolite-4 eluted at 15.64. |  |
| Table S3: sequence analysis of genes predicted within the putative caryophyllene synthase biosynthetic cluster. |  |
| Figure S16: Infrared (IR) spectrum compound **1.** |  |
| Figure S17: ^1^H NMR spectrum (CDCl_3_, 500 MHz) of **1** isolated from *A. oryzae* contains two transgenic genes (Hfas-CS and FAD-redox) of *H. fasciculare*. |  |
| Figure S18: ^13^C NMR spectrum (CDCl_3_, 500 MHz) of **1**. |  |
| Figure S19: COSY spectrum (CDCl_3_, 500 MHz) of **1**. |  |
| Figure S20: HSQC spectrum (CDCl_3_, 500 MHz) of **1.** |  |
| Figure S21: HMBC spectrum (CDCl_3_, 500 MHz) of **1**. |  |
| Figure S22: NOESY spectrum (CDCl_3_, 500 MHz) of **1**. |  |
| Figure S23: High resolution mass (HRMS) of purified compound **1** |  |
| Figure S24: Infrared (IR) spectrum compound **2.** |  |
| Figure S25: Compound 2 chemical structure and key NOESY correlations. |  |
| Figure S26: ^1^H NMR spectrum (CDCl_3_, 500 MHz) of **2**. |  |
| Figure S27: ^13^C NMR spectrum (CDCl_3_, 500 MHz) of **2** |  |
| Figure S28: COSY spectrum (CDCl_3_, 500 MHz) of **2**. |  |
| Figure S29: HSQC spectrum (CDCl_3_, 500 MHz) of **2**. |  |
| Figure S30: HMBC spectrum (CDCl_3_, 500 MHz) of **2**. |  |
| Figure S31: NOSY spectrum (CDCl_3_, 500 MHz) of **2** |  |
| Figure S32: Infrared (IR) spectrum compound **3**. |  |
| Figure S33: Compound 3 chemical structure and key NOESY correlations. |  |
| Figure S34: ^1^H NMR spectrum (CDCl_3_, 500 MHz) of **3**. |  |
| Figure S35: ^13^C NMR spectrum (CDCl_3_, 500 MHz) of **3.** |  |
| Figure S36: COSY spectrum (CDCl_3_, 500 MHz) of **3**. |  |
| Figure S37: HSQC spectrum (CDCl_3_, 500 MHz) of **3**. |  |
| Figure S38: HMBC spectrum (CDCl_3_, 500 MHz) of **3**. |  |
| Figure S39: NOESY spectrum (CDCl_3_, 500 MHz) of **3**. |  |
| Figure S40: Infrared (IR) spectrum compound **4.** |  |
| Figure S41: ^1^H NMR spectrum (CDCl_3_, 500 MHz) of **4**. |  |
| Figure S42: ^13^C NMR spectrum (CDCl_3_, 500 MHz) of **4**. |  |
| Figure S43: COSY spectrum (CDCl_3_, 500 MHz) of **4.** |  |
| Figure S44: HSQC spectrum (CDCl_3_, 500 MHz) of **4**. |  |
| Figure S45: HMBC spectrum (CDCl_3_, 500 MHz) of **4**. |  |
| Figure S46: NOESY spectrum (CDCl_3_, 500 MHz) of 4. |  |
| Table S4. ^1^H (500 MHz) and ^13^C (125 MHz) NMR Data*^a^* of 2 and 3. |  |
| Table S5. ^1^H (500 MHz) and ^13^C (125 MHz) NMR Data*^a^* of 1 and 4. |  |
| Figure S47: Amino acids sequence alignment of Hfas-CS, Hfas-94A, Hfas-94B, Omp-6, Omp-7. |  |
| Figure S48: A schematic diagram shows the construction of *A. oryzae* expression vectors. |  |
| Figure S49: Restriction digestion analysis using *EcoR*V, showing the correct construction of *A. oryzae* expression plasmids. |  |
| Table S6: primers used for yeast recombination and plasmids verifications |  |
| Figure S50: Schematic representation of dual gene plasmid construction. |  |

**Methods**

**GC-MS sampling**

Samples analysis was carried out using PerkinElmer Autosystem Gas Chromatography-Mass Spectroscopy system was used to analyse volatile metabolites of *A. oryzae* mutants. Electron impact at 70 eV, HP-5 MS quartz capillary column (30 m x 0.25 mm, 0.25 µm film thickness) with helium (purity 99.999%) as carrier gas at a flow rate of 1.0 ml/minutes were the parameters used. Experimental conditions for GC analysis of extract were: temperature of injection = 280 °C, temperature of column oven = 80 °C for 2 minutes and set to run at 8 °C/minutes to 280 °C (this was kept constant at 280 °C for 3 minutes), injection volume = 1 µl. The split ratio was adjusted at 20:1. Mass fragmentation of isolated compounds, were recorded within a spectrum ranged from 20-800. Isolated peaks were then submitted to NIST08 database for identification.

**RNA extraction**

For RNA extraction of *H. fasciculare,* the TRIzol method (1) was modified, where one marginal plug of a 14 day growing mycelia on a MEA (15 g/L malt extract, agar 15 g/L) plate, was inoculated into 100 ml of MEB (15 g/L malt extract) medium and incubated at 25°C and 200 rpm for 21 days. Growing mycelia was collected from the culture, and flash frozen in liquid nitrogen, to be incubated at -80°C for 1 hour, and then freeze dried. Dried mycelia grounded in the presence of liquid nitrogen, and 50 mg of it was added to a 1.5 ml Eppendorf tube containing 1 ml of TRIzol reagent, and vortexed until no clumps seen. Tubes were left at room temperature for 30 minutes, then centrifuged for 5 minutes at 12000 rpm. Supernatants were transferred into new Eppendorf tubes, where 200 µl of chloroform was added to each tube, and vortexed vigorously. The tubes were then centrifuged at 12000 rpm, 4°C for 15 minutes. The aqueous layer was transferred to new Eppendorf containing 500 µl of ice-cold isopropanol and incubated at room temperature for 20 minutes. The tubes were then centrifuged at 12000 rpm, 4°C for 10 minutes. Supernatants were discarded, and pellets washed with ice-cold 70% ethanol, and centrifuged for 2 minutes. Pellets were then air dried and dissolved in 30 µl of DEPC treated water. To check the integrity of the resulted RNA, 2-5 µl of it was visualised on 1% of agarose gel. The RNA was then purified using RNase-free DNase I kit (Qiagen). 1 µl of 10x reaction buffer containing MgCl2 and 1 µl of DNase I were added to 1 µg of the prepared RNA, and reaction was made up to 10 µl with DEPC water. Tubes were then incubated at 37°C for 90 minutes. 1 µl of EDTA was added to each tube and incubated at 65°C for 15 minutes. For cDNA synthesis, the first strand cDNA Kit (Thermo Scientific) was used. 1 µl of Oligo (dT)^18^ primer was added to 1 µg of RNA, and DEPC treated water added to final volume of reaction 11 µl, and incubated for 5 minutes at 65°C, then reaction chilled on ice for 2 minutes. The following amounts of reagents; 5x Reaction Buffer (4 µl), RiboLock RNase Inhibitor (1 µl), 10 mM dNTPs (2 µl) and M-MuLV Reverse Transcriptase (2 µl) were added to each reaction tube, mixed, and incubated at 37°C for 60 minutes. The reaction was then terminated by incubating at 70°C for 5. The synthesised cDNA was then used as a template to amplify the full length of genes encoding terpene synthase.

***A. oryzae* mutants**

cDNA synthesis for genes; Hfas-terp255, Hfas-terp94A, Hfas-terp94B, Hfas-terp105, Hfas-terp147, Hfas-terp804, Hfas-terp342, Hfas-terp179, and Hfas-terp344, was therefore carried out as described above.

However, of all selected terpene genes, the encoding region of only Hfas-terp94A, Hfas-terp94B, Hfas-terp179 and Hfas-terp344 could be fully synthesised from *H. fasciculare* cDNA. Following the manufacture protocol of pJET1.2 cloning, the cDNA version of each gene, was cloned into pJET1.2 cloning vector, which then transformed to *E. coli* (strain DH5α). Using Phusion High Fidelity kit, all genes were PCR amplified from their pJET vectors. The plasmid backbone pTYGSarg was digested by *Asc*I enzyme, and the region between *Padh* and *Teno* was removed through gel purification, each Hfterp-gene was independently incorporated in the backbone with the 3’ end region of *adh* promoter and the 5’ end region of *eno* terminator (Figure S48 and S49 supplementary information) through a classical yeast homologous recombination protocol (2). The adapted protocol from (3) was used to protoplast mediate *A. oryzae* (NSARI) with one of the produced expression vector, leading to a population of NSARI mutants. Five randomly selected potential transformants for each gene were subcultured three rounds on selective plates (Czapex-Dox agar lacking arginine). Polymerase chain reaction of the integrated genes from all *Aspergillus* putative mutants with either gene specific or yeast recombination primers (Table S8 supplementary information), of predicted size of amplicons indicated successful transformation in two transformants of NSAR1-344, one transformatant of NSAR1-Hf94A, two transformants of NSAR1-94B, one transformant of NSAR1-179, two transformants of NSAR1-Omp-6 and four transformants of NSAR1- Hf255 were confirmed by PCR reactions. No pcr amplicon could be obtained for NSAR1-Omp-7 transformation experiments.

**Production of dual-gene mutant strains**

Our heterologous expression of *H. fasciculare* terpene synthase enzymes in *A. oryzae*, has characterized Hfas-344 as caryophyllene synthase, and to better our knowledge about the genes involved in naematolin biosynthetic genes, we constructed a plasmid contain both caryophyllene synthase and the gene nearby; FAD-redox. FAD-redox was pcr amplified from Hfas cDNA, and caryophyllene synthase was amplified from its expression vector pTYGS-caryo-arg. The two fragments were then homologous recombined in Y10000 S. *cerevisiae* to construct the dual gene expression vector using the backbone pTYGS-ade. The caryophyllene synthase was located between the *adh* promoter and terminator of *A. oryzae* and the FAD-redox located between *gpd* promoter and *eno* terminator of *A. nidulans* (Figure S50).

We then deployed the (3) method to transfer the correctly assembled expression vector to *A. oryzae* (NSARI). Out of five randomly selected transformants, two have shown the expected pcr amplicons for both transgenes.


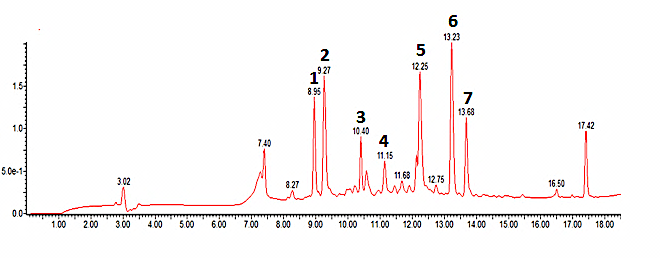


Figure S1: LC-MS chromatogram (UV absorbance/retention time) of *H. fasciculare* (CGC culture) crude extract, identifying 7 main peaks including naematolin (peak 5).


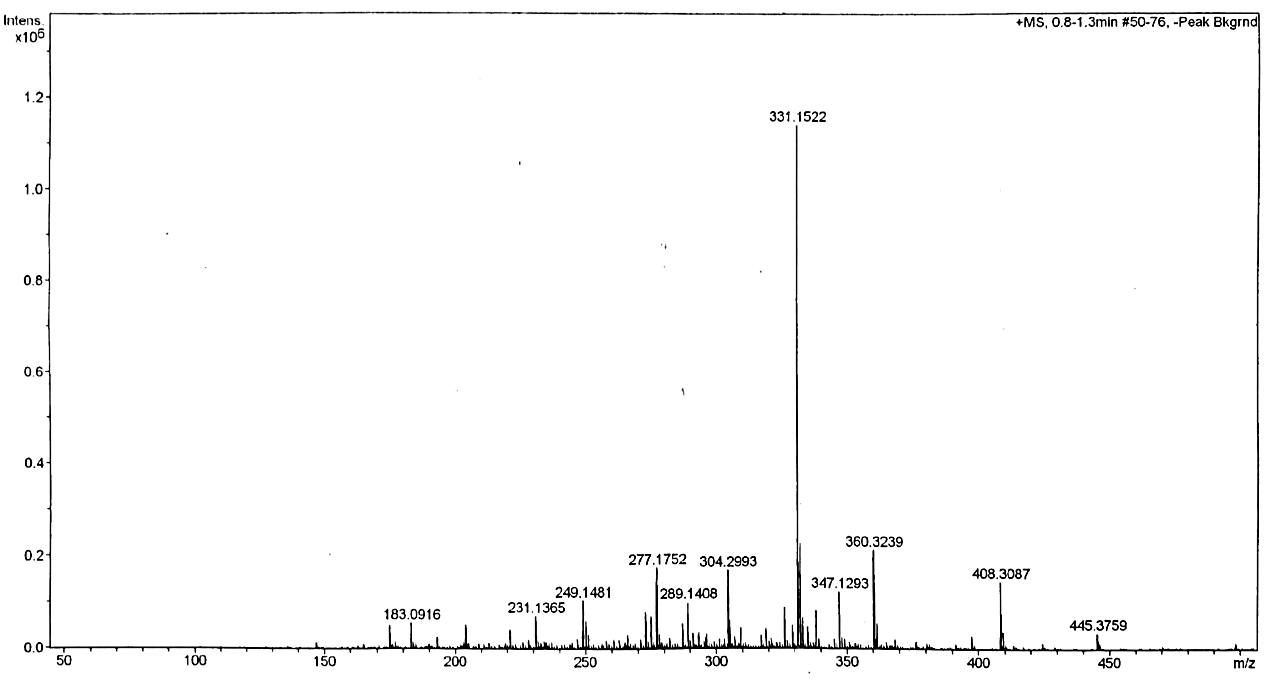


Figure S2: High resolution mass (HRMS) of purified naematolin showing a major ion isotope of m/z 331.1522 for C_17_H_24_NaO_5_.


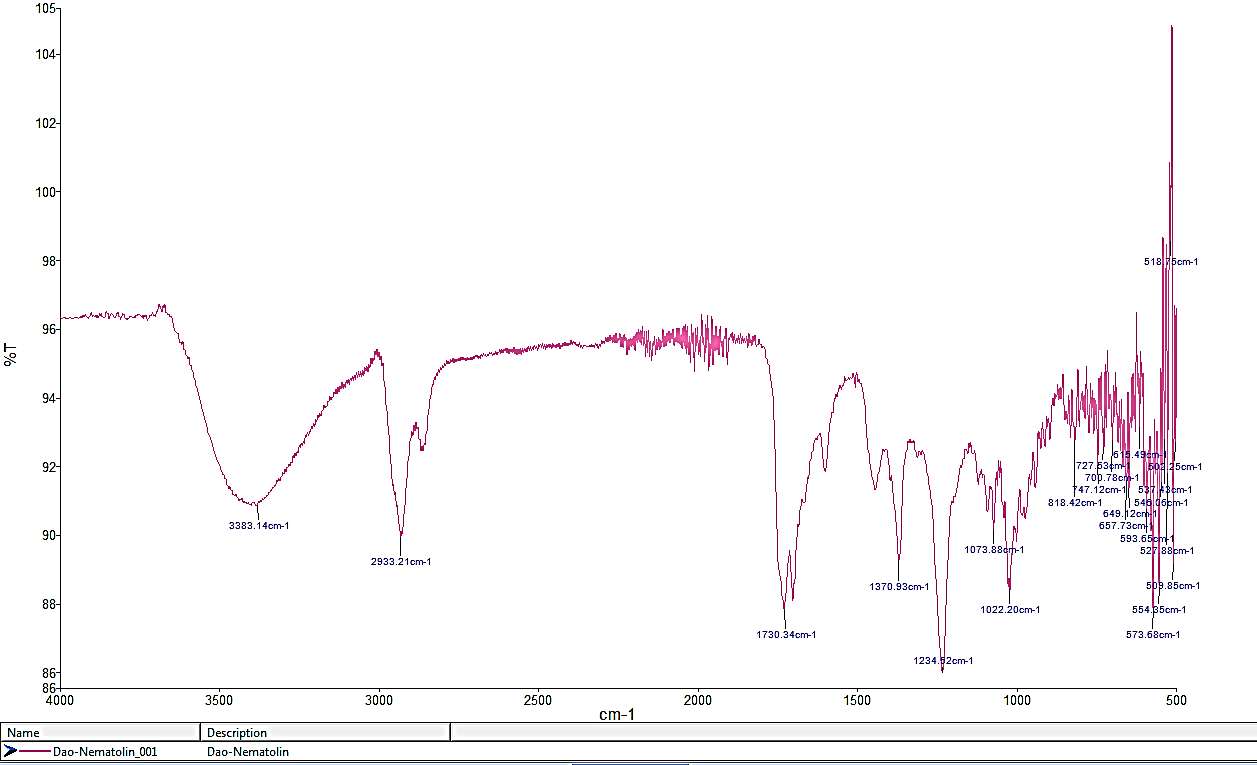


Figure S3: Naematolin infrared (IR) spectrum.

Figure S4: Naematolin predicted chemical structure.

Figure S5: ^1^H NMR spectrum (CDCl_3_, 125 MHz) of naematolin.

Figure S6: ^13^C NMR (500 MHz) spectrum (CDCl_3_, 125 MHz) of Naematolin.

Figure S7: COSY (500 MHz) spectrum of naematolin.

Figure S8: HSQC (500 MHz) spectrum of Naematolin.

Figure S9: HMBC (500 MHz) spectrum of Naematolin.

Table S1: Carbon and proton chemical shift position for naematolin of *H. sublateritium* (PDB culture extract) and NMR reference data for naematolin (Backens *et al.,* 1984), showing significant match with the NMR data of reported naematolin derivative.

| **No. of C** | **Observed (naematolin)** | | **Reference naematolin isomer (Backens *et al.,* 1984)** | |
| --- | --- | --- | --- | --- |
|  | **C (ppm)** | **H (ppm)** | **C (ppm)** | **H (ppm)** |
| 1 | 46.7 | 2.85 (d, *J* = 10.9 Hz, 1H) | 46.7 | 2.90 d |
| 2 | 71.7 | 3.68 (dd, *J* = 11.3, 2.5 Hz, 1H) | 71.7 | 3.61 dd |
| 3 | 77.6 | 4.15 (d, *J* = 2.4 Hz, 1H) | 77.5 | 4.04 d |
| 4 | 146.9 | - | 146.9 | - |
| 5 | 116.8 | 6.28 (d, *J* = 9.1 Hz, 1H) | 116.7 | 6.29 d |
| 6 | 71.2 | 5.79 (s, 1H) | 71.6 | 5.79 d |
| 7 | 196.2 | - | 196.2 | - |
| 8 | 149.4 | - | 149.4 | - |
| 9 | 35.2 | 3.09 (td, *J* = 10.5, 5.2 Hz, 1H) | 35.2 | 3.26 td |
| 10 | 41.0 | 2.14 (m, 1H) | 40.9 | 2.13 dd |
|  |  | 1.79 (m, 1H) |  | 1.77 m |
| 11 | 35.1 | - | 35.0 | - |
| 12 | 23.8 | 1.21 (s, 3H) | 23.8 | 1.16 s |
| 13 | 33.7 | 1.27 (s, 3H) | 33.5 | 1.26 s |
| 14 | 18.8 | 1.55 (s, 3H) | 18.6 | 1.48 s |
| 15 | 126.5 | 6.74 (s, 1H) | 126.4 | 6.35 s |
|  |  | 5.76 (s, 1H) |  | 5.74 s |
| 16 | 170.3 | - | 170.3 | - |
| 17 | 20.9 | 2.19 (s, 3H) | 20.8 | 2.08 s |


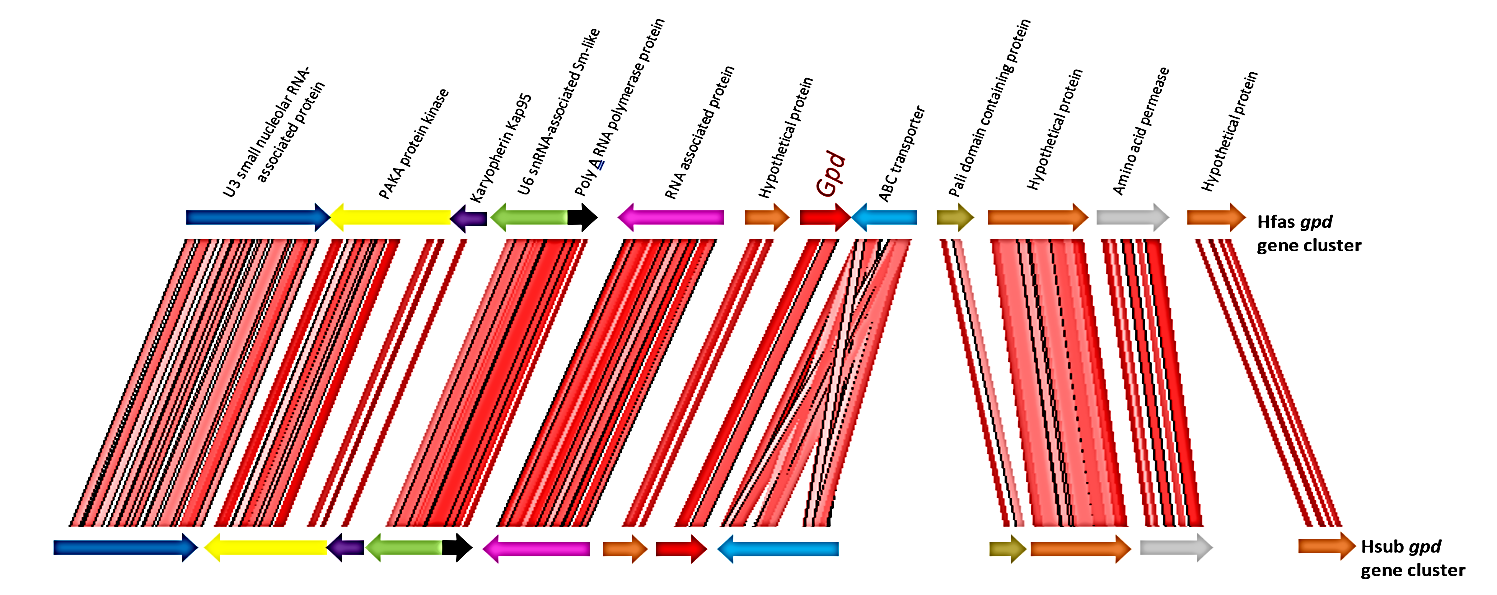


Figure S10: A comparison of the *gpd* gene region from *H. fasciculare* and the *gpd* gene region from *H. sublateritium* using Artemis Comparison Tool (ACT). Putative gene annotation is indicated. The red bars indicative of high similarity (80-100%) of sequences and orientation.


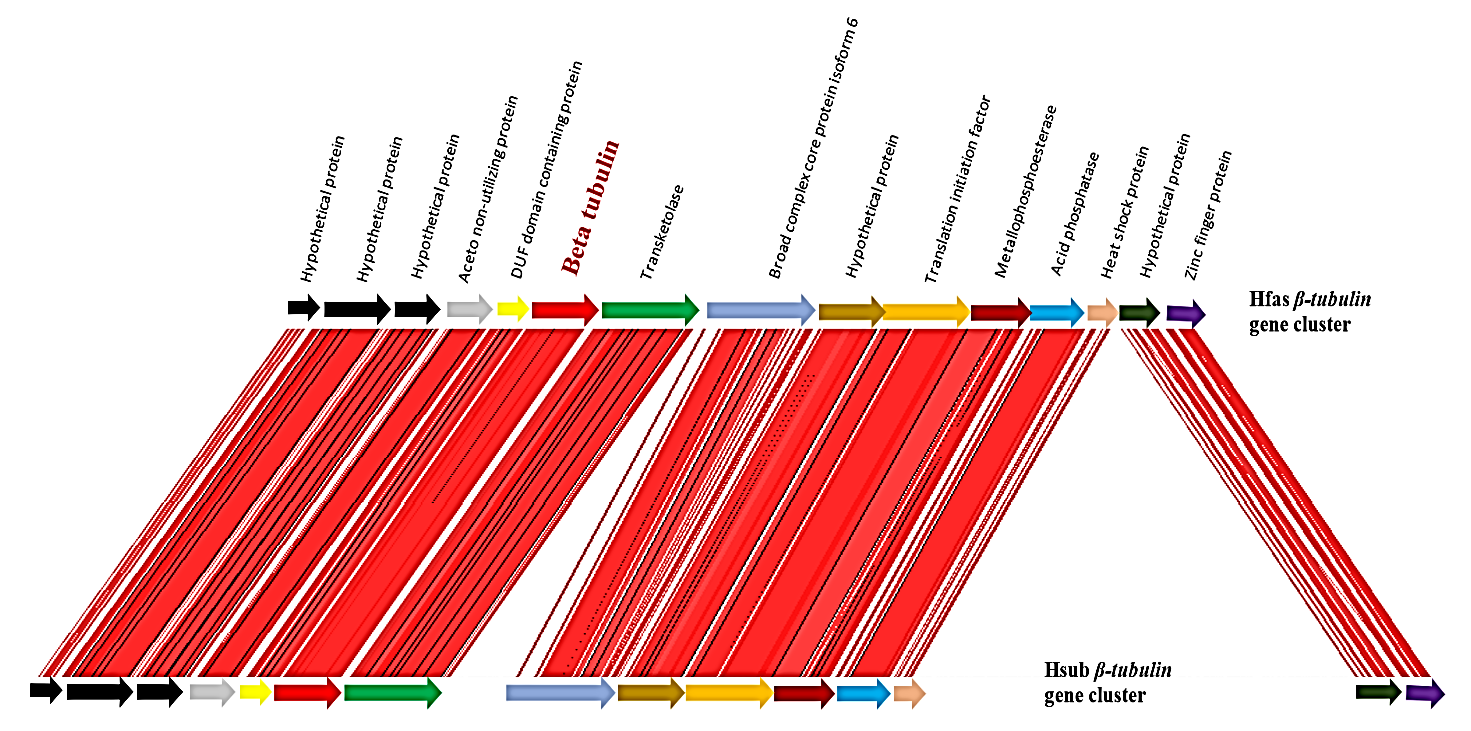


Figure S11: A comparison of the *β-tubulin* gene region from *H. fasciculare* and *H. sublateritium* using Artemis Comparison Tool (ACT). Putative gene function is indicated upstream the annotated exons. The red bars indicative of high similarity (80-100%) of sequences, and orientation

**Table S2: Genome assembly statistic of both *H. sublateritium and H. fasciculare***

| **Genome assembly parameter** | ***H. sublateritium*** | ***H. fasciculare*** |
| --- | --- | --- |
| Genome assembly size (Mbp) | 48.03 | 58.84 |
| Number of contigs | 1329 | 3400 |
| Largest contig bp | 1730000 | 408079 |
| N50 | 44000 | 49633 |
| Predicted genes | 17911 | Gene ˃300 bp = 23038 |

Figure S12: Shows mass spectrum comparison of NSAR1-Hfas344 transformant-4 of metabolite-1 eluted at 12.12 (A) and caryophyllene the mass spectrum of best hit from Nist database (B).


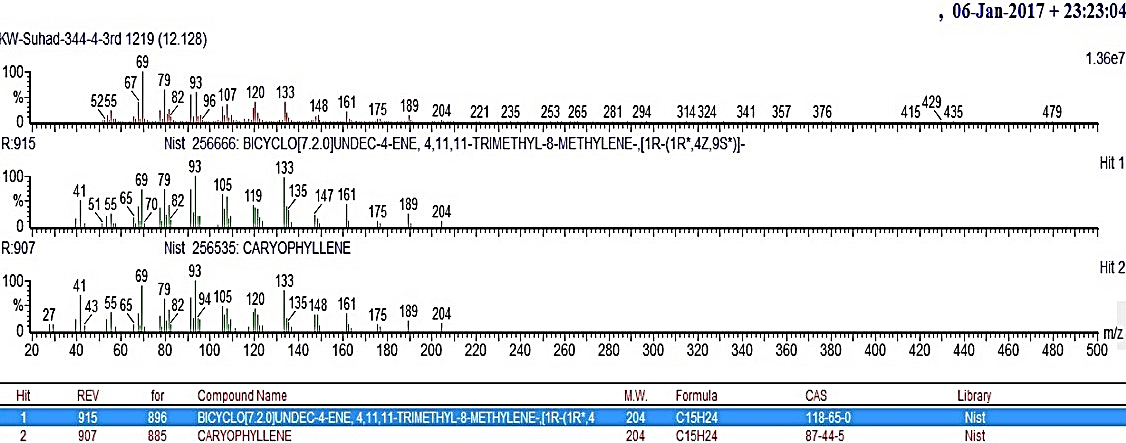

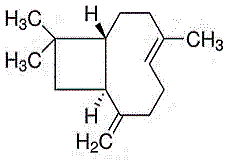


Figure S13: Shows mass spectrum comparison of NSAR1-Hfas344 transformant-4 of metabolite-2 eluted at 14.18 (A) and caryophyllene the mass spectrum of best hit from Nist database (B).


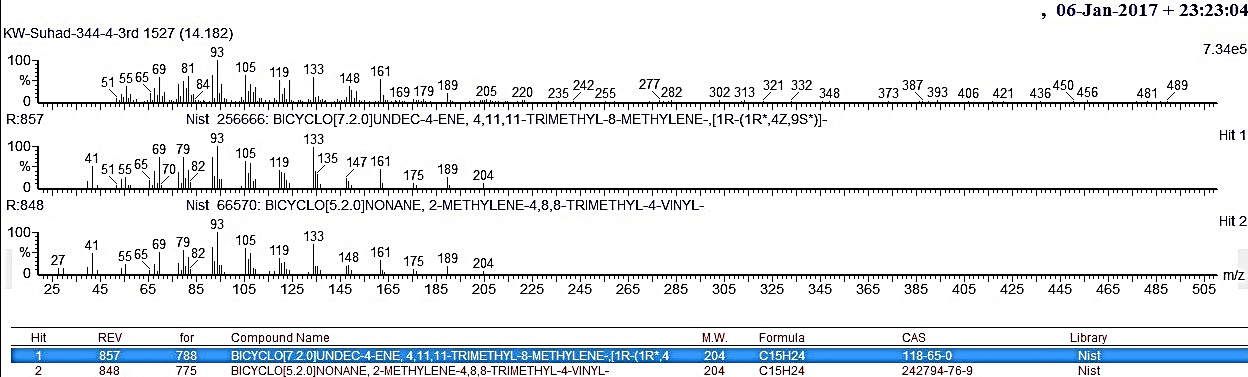

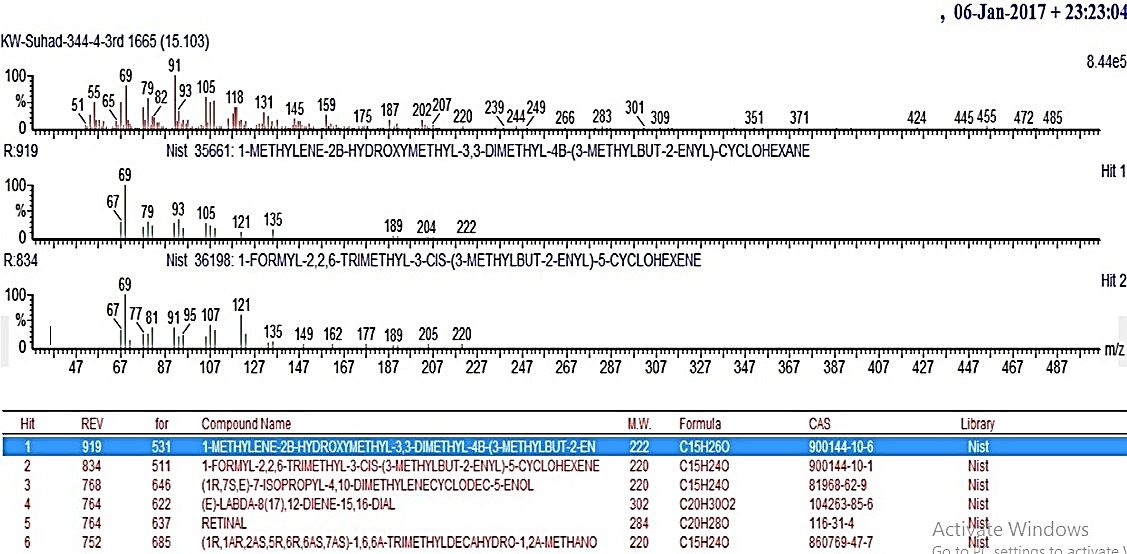


Figure S14: Shows mass spectrum comparison of NSAR1-Hfas344 transformant-4 of metabolite-3 eluted at 15.10 (A) and the best hit from Nist database (B).


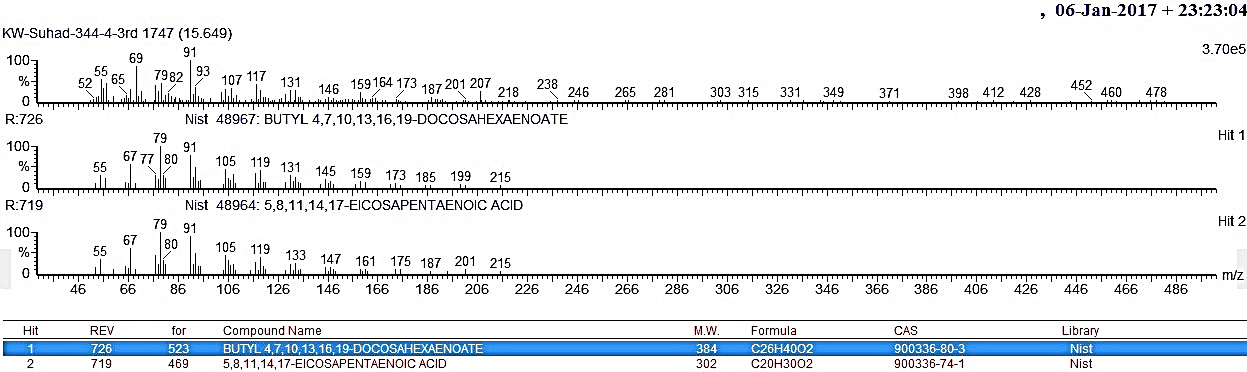


Figure 15: Shows mass spectrum comparison of NSAR1-Hfas344 transformant-4 of metabolite-4 eluted at 15.64 (A) and the best hit from Nist database (B).

Table S3: sequence analysis of genes predicted within the putative caryophyllene synthase biosynthetic cluster. Gene function was predicted using BLAST search (NCBI and JGI).

| Gene predicted function (*H. fasciculare* gene cluster 344 and 128 ) | Gene predicted function (*H. sublateritium* gene cluster 99) | Homology % |
| --- | --- | --- |
| Hypothetical protein | Hypothetical protein | 86% |
| Aldo-keto-reductase | Aldo-keto-reductase | 87% |
| RNA-directed RNA plymerase | RNA-directed RNA polymerase | 87% |
| Zinc metalopeptidase | Zinc metallopeptidase | 88% |
| Transcription factor | Transcription factor | 90% |
| Terpene synthase | Terpene synthase | 88% |
| FAD oxidoreductase | FAD oxidoreductase | 87% |
| Dimeric alpha-beta protein | Dimeric alpha-beta protein | 87% |
| Cytochrome P450-1 | Cytochrome P450-1 | 90% |
| Cytochrome P450-2 | Cytochrome P450-2 | 88% |
| Hypothetical protein | Hypothetical protein | 83% |
| SDR | SDR | 86% |
| Zinc alcohol dehydrogenase | Zinc alcohol dehydrogenase | 84% |
| Cytochrome P450-3 | Cytochrome P450-3 | 90% |
| Hypothetical protein | Hypothetical protein | 86% |


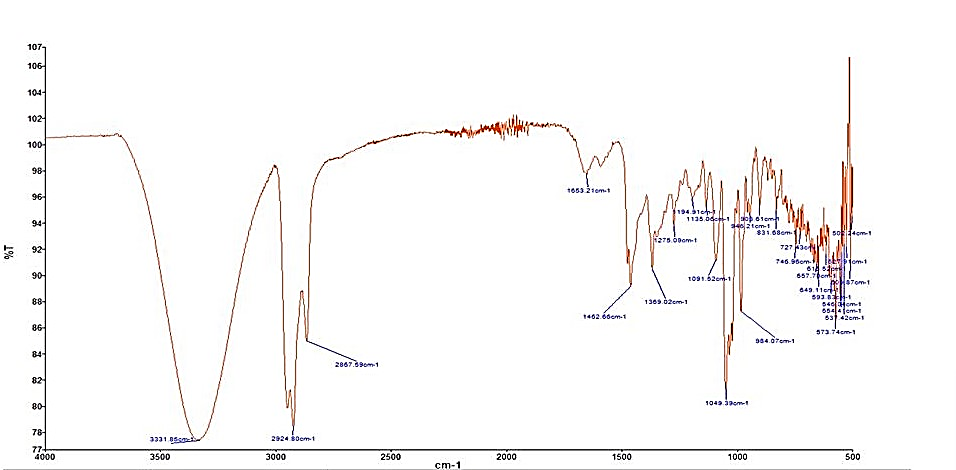


Figure S16: Infrared (IR) spectrum compound **1**.

Figure S17: ^1^H NMR spectrum (CDCl_3_, 500 MHz) of **1** isolated from *A. oryzae* contains two transgenic genes (Hfas-CS and FAD-redox) of *H. fasciculare*.

Figure S18: ^13^C NMR spectrum (CDCl_3_, 500 MHz) of **1**.

Figure S19: COSY spectrum (CDCl_3_, 500 MHz) of **1**.

Figure S20: HSQC spectrum (CDCl_3_, 500 MHz) of **1**.

Figure S21: HMBC spectrum (CDCl_3_, 500 MHz) of **1**.

Figure S22: NOESY spectrum (CDCl_3_, 500 MHz) of **1**.

**Compound 2:** colorless oil; [α] – 23.6º (*c* 0.1, CHCl_3_); IR *ν*_max_ 3350, 2926, 1448, 1034 cm^–1^; δ_H_ (500 MHz, CDCl_3_) 0.83 (1H, m, H-3a), 0.91 (3H, s, H-12), 1.20 (3H, s, H-13), 1.54 (2H, m, H-2), 1.66 (1H, m, H-10a), 1.76 (1H, m, H-10b), 1.93 (1H, m, H-7a), 2.09 (1H, m, H-1), 2.51 (1H, m, H-3b), 2.86 (1H, d, *J* = 10.0, H-5), 2.94 (1H, m, H-7b), 3.08 (1H, m, H-9), 3.50 (1H, d, *J* = 15.0 Hz, H-14a), 3.77 (1H, d, *J* = 15.0 Hz, H-14b), 3.80 (1H, m, H-6), 5.02 (1H, br s, H-15a), 5.03 (1H, br s, H-15b); δ_C_ (125 MHz, CDCl_3_) 21.0 (C-2), 25.2 (C-12), 29.7 (C-13), 33.3 (C-11), 35.7 (C-3), 38.0 (C-10), 42.2 (C-9), 45.4 (C-7), 52.9 (C-1), 62.9 (C-14), 63.4 (C-4), 66.4 (C-5), 69.3 (C-6), 116.1 (C-15), 144.7 (C-8); HRESIMS *m/z* 275.1625 [M + Na]^+^ (calcd for C_15_H_24_O_3_Na, 275.1623).


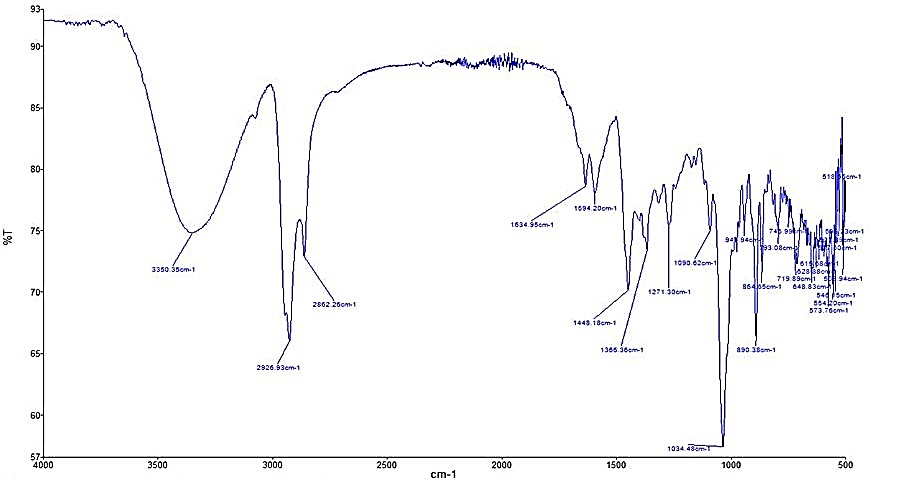


Figure S24: Infrared (IR) spectrum compound **2**.


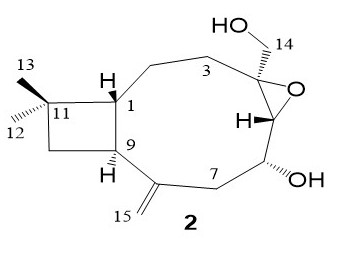

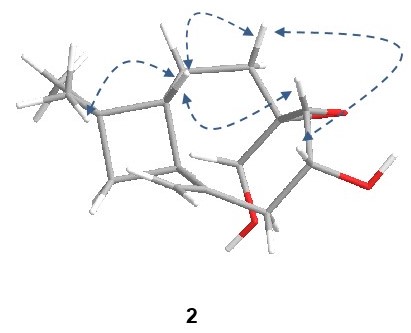


Figure S25: Compound 2 chemical structure and key NOESY correlations.

Figure S26: ^1^H NMR spectrum (CDCl_3_, 500 MHz) of **2**.

Figure S27: ^13^C NMR spectrum (CDCl_3_, 500 MHz) of **2**.

Figure S28: COSY spectrum (CDCl_3_, 500 MHz) of **2**.

Figure S29: HSQC spectrum (CDCl_3_, 500 MHz) of **2**.

Figure S30: HMBC spectrum (CDCl_3_, 500 MHz) of **2**.

Figure S31: NOSY spectrum (CDCl_3_, 500 MHz) of **2**.

Comp-3

**Compound 3:** colorless oil; [α] – 38.4º (*c* 0.15, CHCl_3_); IR *ν*_max_ 3325, 2972, 1379, 1046 cm^–1^; δ_H_ (500 MHz, CDCl_3_) 0.90 (3H, s, H-12), 1.21 (3H, s, H-13), 1.48 (2H, m, H-2), 1.50 (1H, m, H-10a), 1.70 (1H, m, H-10b), 1.78 (1H, m, H-7a), 1.80 (1H, m, H-3a), 2.11 (1H, m, H-1), 2.52 (1H, m, H-3b), 2.87 (1H, m, H-7b), 2.93 (1H, m, H-9), 3.88 (1H, d, *J* = 12.5 Hz, H-14a), 4.18 (1H, d, *J* = 12.5 Hz, H-14b), 4.62 (1H, m, H-6), 4.80 (1H, br s, H-15a), 4.81 (1H, br s, H-15b), 5.20 (1H, d, *J* = 10.5 Hz, H-5); δ_C_ (125 MHz, CDCl_3_) 25.6 (C-12), 26.0 (C-2), 29.6 (C-13), 33.3 (C-11), 36.3 (C-3), 37.7 (C-10), 42.9 (C-9), 48.3(C-7), 52.3 (C-1), 61.6 (C-14), 68.7 (C-6), 113.7 (C-15), 128.2 (C-5), 139.7 (C-4), 148.0 (C-8); HRESIMS *m/z* 259.1675 [M + Na]^+^ (calcd for C_15_H_24_O_2_Na, 259.1674).


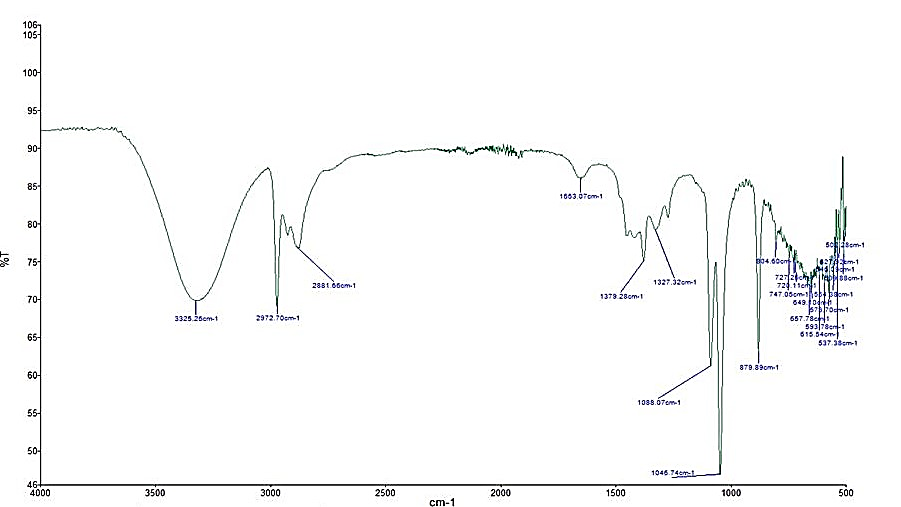


Figure S32: Infrared (IR) spectrum compound **3**.


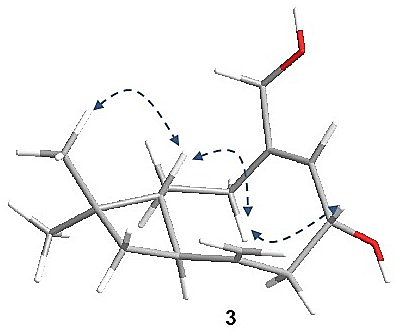

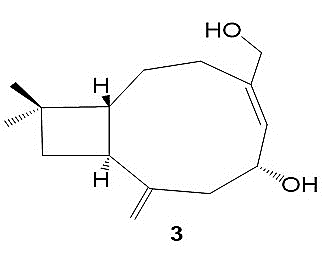


Figure S33: Compound 3 chemical structure and key NOESY correlations.

Figure S34: ^1^H NMR spectrum (CDCl_3_, 500 MHz) of **3**.

Figure S35: ^13^C NMR spectrum (CDCl_3_, 500 MHz) of **3**.

Figure S36: COSY spectrum (CDCl_3_, 500 MHz) of **3**.

Figure S37: HSQC spectrum (CDCl_3_, 500 MHz) of **3**.

Figure S38: HMBC spectrum (CDCl_3_, 500 MHz) of **3**.

Figure S39: NOESY spectrum (CDCl_3_, 500 MHz) of **3**.


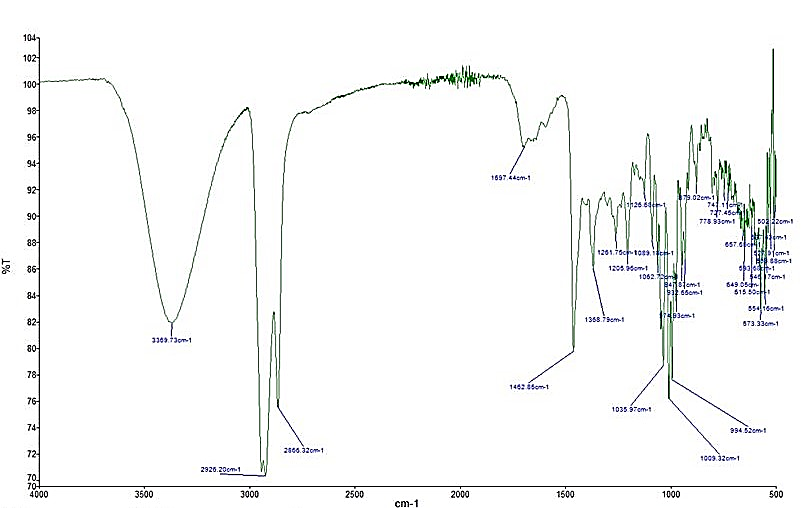


Figure S40: Infrared (IR) spectrum compound **4**.

Figure S41: ^1^H NMR spectrum (CDCl_3_, 500 MHz) of **4**.

Figure S42: ^13^C NMR spectrum (CDCl_3_, 500 MHz) of **4**.

Figure S43: COSY spectrum (CDCl_3_, 500 MHz) of **4**.

Figure S44: HSQC spectrum (CDCl_3_, 500 MHz) of **4**.

Figure S45: HMBC spectrum (CDCl_3_, 500 MHz) of **4**.

Figure S46: NOESY spectrum (CDCl_3_, 500 MHz) of **4**.

**Table S4.** ^1^H (500 MHz) and ^13^C (125 MHz) NMR Data*^a^* of **2** and **3**

| Position | **2** | |  | **3** | | |
| --- | --- | --- | --- | --- | --- | --- |
|  | δ_H_ mult. (*J* in Hz) | δ_C_ |  | δ_H_ mult. (*J* in Hz) | δ_C_ |  |
|  |  |  |  |  |  |  |
| 1 | 2.09 m | 52.9 |  | 2.11 m | 52.3 |  |
| 2 | 1.54 m | 21.0 |  | 1.48 m | 26.0 |  |
|  |  |  |  |  |  |  |
| 3 | 2.51 m | 35.7 |  | 2.52 m | 36.3 |  |
|  | 0.83 m |  |  | 1.80 m |  |  |
| 4 |  | 63.4 |  |  | 139.7 |  |
| 5 | 2.86 d (10.0) | 66.4 |  | 5.20 d (10.5) | 128.2 |  |
| 6 | 3.80 m | 69.3 |  | 4.62 m | 68.7 |  |
| 7 | 2.94 m | 45.4 |  | 2.87 m | 48.3 |  |
|  | 1.93 m |  |  | 1.78 m |  |  |
| 8 |  | 144.7 |  |  | 148.0 |  |
| 9 | 3.08 m | 42.2 |  | 2.93 m | 42.9 |  |
| 10 | 1.76 m | 38.0 |  | 1.70 m | 37.7 |  |
|  | 1.66 m |  |  | 1.50 m |  |  |
| 11 |  | 33.3 |  |  | 33.3 |  |
| 12 | 0.91 s | 25.2 |  | 0.90 s | 25.6 |  |
| 13 | 1.20 s | 29.7 |  | 1.21 s | 29.6 |  |
| 14 | 3.77 d (15.0) | 62.9 |  | 4.18 d (12.5) | 61.6 |  |
|  | 3.50 d (15.0) |  |  | 3.88 d (12.5) |  |  |
| 15 | 5.03 br s | 116.1 |  | 4.81 br s | 113.7 |  |
|  | 5.02 br s |  |  | 4.80 br s |  |  |
|  |  |  |  |  |  |  |

*^a^* Recorded in CDCl_3_.

**Table S5.** ^1^H (500 MHz) and ^13^C (125 MHz) NMR Data*^a^* of **1** and **4**

| Position | **1** | |  | **4** | | |
| --- | --- | --- | --- | --- | --- | --- |
|  | δ_H_ mult. (*J* in Hz) | δ_C_ |  | δ_H_ mult. (*J* in Hz) | δ_C_ |  |
|  |  |  |  |  |  |  |
| 1 | 1.90 m | 47.4 |  | 1.77 m | 48.9 |  |
| 2 | 1.61 m | 20.9 |  | 1.78 m | 22.1 |  |
|  | 1.45 m |  |  | 1.44 m |  |  |
| 3 | 1.48 m | 37.7 |  | 1.50 m | 36.1 |  |
|  | 1.20 m |  |  | 0.98 m |  |  |
| 4 |  | 37.0 |  |  | 37.6 |  |
| 5 | 3.28 d (8.0) | 82.3 |  | 3.33 m | 74.1 |  |
| 6 | 3.95 m | 73.7 |  | 1.82 m | 27.0 |  |
|  |  |  |  | 1.68 m |  |  |
| 7 | 1.77 m | 42.8 |  | 1.85 m | 35.7 |  |
|  |  |  |  | 1.44 m |  |  |
| 8 |  | 72.4 |  |  | 72.7 |  |
| 9 | 2.57 m | 42.1 |  | 2.46 m | 41.6 |  |
| 10 | 1.75 m | 32.6 |  | 1.74 m | 32.6 |  |
|  | 1.50 m |  |  | 1.62 m |  |  |
| 11 |  | 33.3 |  |  | 34.0 |  |
| 12 | 0.95 s | 24.5 |  | 0.93 s | 24.5 |  |
| 13 | 1.19 s | 29.4 |  | 1.18 s | 28.7 |  |
| 14 | 0.96 s | 25.5 |  | 1.00 s | 30.2 |  |
| 15 | 1.80 d (14.0) | 36.9 |  | 1.84 d (14.0) | 38.4 |  |
|  | 1.34 d (14.0) |  |  | 1.32 d (14.0) |  |  |
|  |  |  |  |  |  |  |

*^a^* Recorded in CDCl_3_.


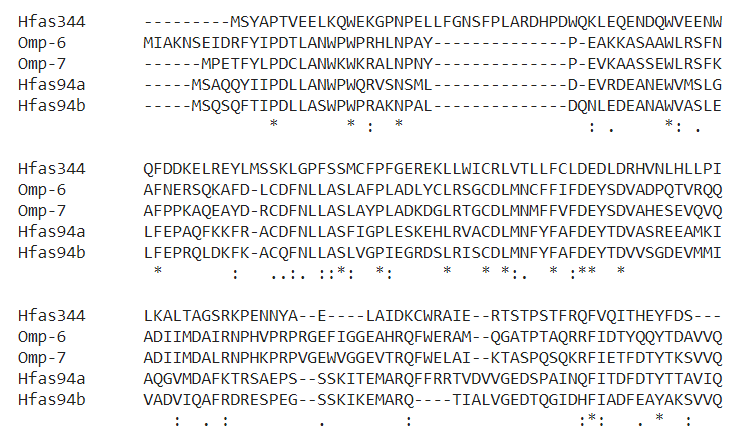

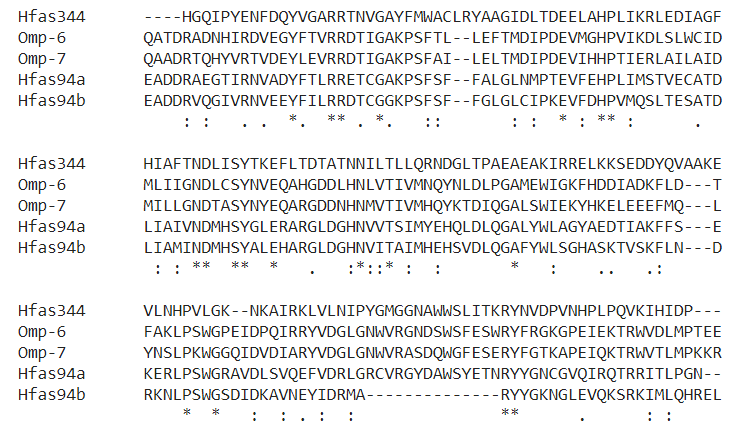

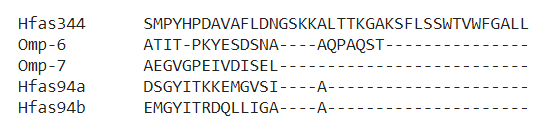


**H-α1-loop (NSE motif)**

**DExxD Motif**

Figure S47: Amino acids sequence alignment of Hfas-CS, Hfas-94A, Hfas-94B, Omp-6, Omp-7.

Blue box = Terpene synthase signature motif D(D/E)xxD.

Yellow box = sesquiterpene synthase motif (NSE/DE).

Red box = conserved residue.


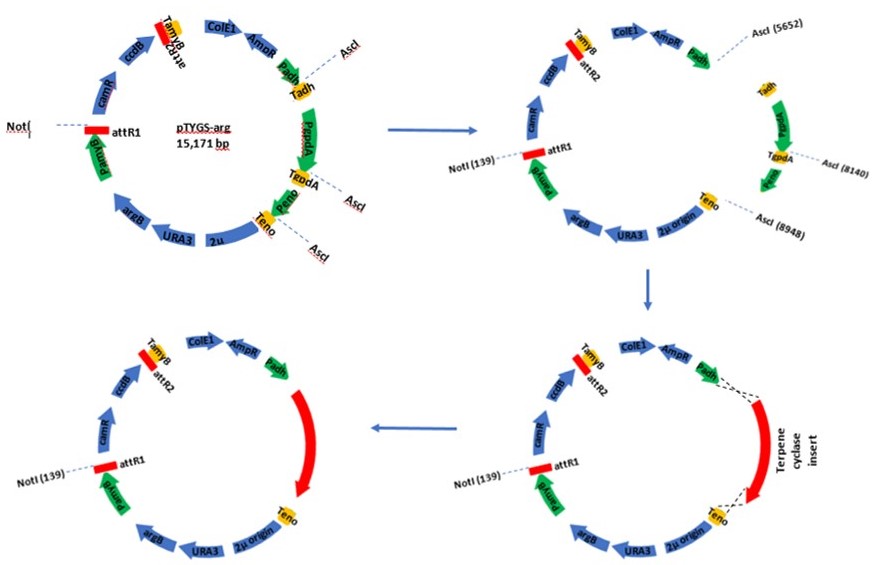


Figure S48: A schematic diagram shows the construction of *A. oryzae* expression vectors. pTYGS-arg backbone was digested with *Asc*I and the region between *Padh* and *Teno* was removed through gel purification.


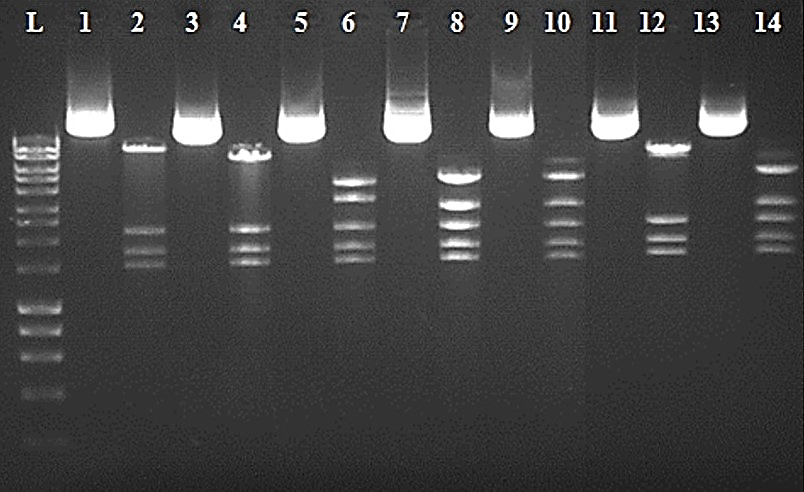


Figure S49: Restriction digestion analysis using *EcoR*V, showing the correct construction of *A. oryzae* expression plasmids. L = 5 *μ*L of hyperladder I. Lane 1 and 2 contain 5 *μ*L of pTYGS-arg-Hf94A miniprep and digestion reaction respectively. Lane 3 and 4 contain 5 *μ*L of pTYGS-arg-Hf94B miniprep and digestion reaction respectively. Lane 5 and 6 contain 5 *μ*L of pTYGS-arg-Hf179 miniprep and digestion reaction respectively. Lane 7 and 8 contain 5 *μ*L of pTYGS-arg-Cop1 miniprep and digestion reaction respectively. Lane 9 and 10 contain 5 *μ*L of pTYGS-arg-Cop3 miniprep and digestion reaction respectively. Lane 11 and 12 contain 5 *μ*L of pTYGS-arg-Omp6 miniprep and digestion reaction respectively. Lane 13 and 14 contain 5 *μ*L of pTYGS-arg-Omp7 miniprep and digestion reaction respectively. All digested plasmids showed the expected bands size.

Table S6: primers used for yeast recombination and plasmids verifications.

| No. | Primer | Sequence (5’->3’) | Fragment size(bp) | Description |
| --- | --- | --- | --- | --- |
| 1 | **Hf-sesqui-94a-F** | TTTCTTTCAACACAAGATCCCAAAGTCAAAATGTCTGCTCAGCAATACATCATTCCTGAT | ±1000 | Amplifying full length of Hfsesqui94A (cDNA) under *adh* promoter and terminator |
| 2 | **Hf-sesqui-94a-R** | GGTTGGCTGGTAGACGTCATATAATCATACCTATGCTATACTGACACCCATCTCTTTCTT |  |  |
| 3 | **Hf-sesqui-94b-F** | TTTCTTTCAACACAAGATCCCAAAGTCAAA ATGTCTCAATCCCAGTTTACTATTCCTGAT | ±1000 | Amplifying full length of Hfsesqui94B (cDNA) under *adh* promoter and terminator |
| 4 | **Hf-sesqui-94b-R** | GGTTGGCTGGTAGACGTCATATAATCATACCTAAGCAGCACCGATTAAGAGCTGGTCTCG |  |  |
| 5 | **Hf-sesqui-344-F** | TTTCTTTCAACACAAGATCCCAAAGTCAAAATGTCGTACGCACCTACCGTCGAAGAGCTC | ±1200 | Amplifying full length of Hfsesqui344(cDNA) under *adh* promoter and terminator |
| 6 | **Hf-sesqui-344-R** | GGTTGGCTGGTAGACGTCATATAATCATAC TTACAACTGCAAACTATACTTCCCGAGGAG |  |  |
| 7 | **344-A*gpd*-F** | AGT TGG ACA GTT TGG TTC GGC GCA CTC CTC GGC GCG CCA GGG AAC ATG TTC ATA ACG CAT | ±2000 | Amplifying A*gpd* promoter |
| 8 | **A*gpd*-R** | GGTGATGTCTGCTCAAGCGGGGTAGCTGTT |  |  |
| 9 | **A*gpd*-FAD-F** | AACAGCTACCCCGCTTGAGCAGACATCACC ATG GCT CTC ACT AAA CGC TTC TCT CTC ATC | ±1464 | Amplifying full length of Hf-FAD-redox (cDNA) under A*gpd* promoter and *eno* terminator |
| 10 | **T*eno*-R** | AGGTTGGCTGGTAGACGTCATATAATCATA |  |  |

Figure S50: Schematic representation of dual gene plasmid construction pTYGS-ade backbone was digested with *Asc*I and the core enzyme (caryophyllene synthase) inserted between *adh* promoter and terminator. The second gene (FAD-redox) was inserted between *gpd* promoter and *eno* terminator.


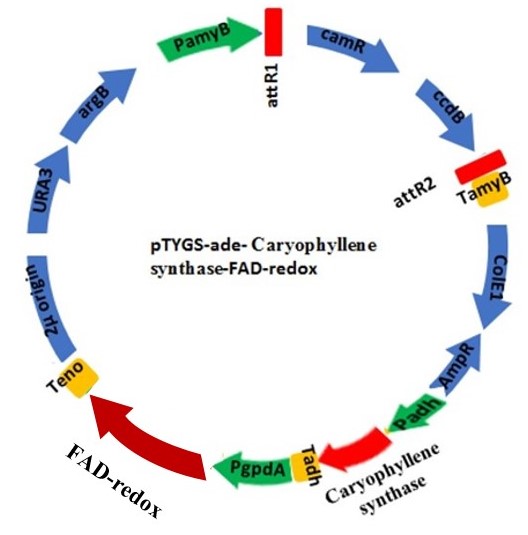


**References**

1- Simms, D., Cizdziel, P.E. and Chomczynski, P. (1993). TRIzol: A new reagent for optimal single-step isolation of RNA. *Focus*, *15*(4), pp.532-535.

2- Gietz, R.D. and Woods, R.A. (2002). Transformation of yeast by lithium acetate/single-stranded carrier DNA/polyethylene glycol method. *Methods in Enzymology*, *350*, pp.87-96.

3- Halo, L.M., Heneghan, M.N., Yakasai, A.A., Song, Z., Williams, K., Bailey, A.M., Cox, R.J., Lazarus, C.M. and Simpson, T.J. (2008). Late stage oxidations during the biosynthesis of the 2-pyridone tenellin in the entomopathogenic fungus *Beauveria bassiana*. *Journal of The American Chemical Society*, *130*(52), pp.17988-17996.
